# 5-Amino-8-hydroxyquinoline-containing Electrospun Materials Based on Poly(vinyl alcohol) and Carboxymethyl Cellulose and Their Cu^2+^ and Fe^3+^ Complexes with Diverse Biological Properties: Antibacterial, Antifungal and Anticancer

**DOI:** 10.3390/polym15143140

**Published:** 2023-07-24

**Authors:** Milena Ignatova, Nevena Manolova, Iliya Rashkov, Ani Georgieva, Reneta Toshkova, Nadya Markova

**Affiliations:** 1 Laboratory of Bioactive Polymers, Institute of Polymers, Bulgarian Academy of Sciences, Acad. G. Bonchev St., Bl. 103A, BG-1113 Sofia, Bulgaria; manolova@polymer.bas.bg; 2 Institute of Experimental Morphology, Pathology and Anthropology with Museum, Bulgarian Academy of Sciences, Acad. G. Bonchev St., Bl. 25, BG-1113 Sofia, Bulgaria; georgieva_any@abv.bg (A.G.); reneta.toshkova@gmail.com (R.T.); 3 Institute of Microbiology, Bulgarian Academy of Sciences, Acad. G. Bonchev St., Bl. 26, BG-1113 Sofia, Bulgaria; markn@bas.bg

**Keywords:** carboxymethyl cellulose, poly(vinyl alcohol), 5-amino-8-hydroxyquinoline, electrospinning, antibacterial, antifungal, anticancer properties

## Abstract

Novel fibrous materials with diverse biological properties containing a model drug of the 8-hydroxyquinoline group—5-amino-8-hydroxyquinoline (5A8Q)—were fabricated using a one-pot method by electrospinning poly(vinyl alcohol) (PVA)/carboxymethyl cellulose (CMC)/5A8Q solutions. Experiments were performed to prepare Cu^2+^ (Fe^3+^) complexes of the crosslinked PVA/CMC/5A8Q materials. The formation of complexes was proven by using scanning electron microscopy (SEM), attenuated total reflection Fourier-transform infrared (ATR-FTIR) spectroscopy, energy-dispersive X-ray spectroscopy (EDX) and X-ray photoelectron spectroscopy (XPS). The release of 5A8Q and 5A8Q.Cu^2+^ (Fe^3+^) was studied and their in vitro release profiles were mostly impacted by the hydrophilic/hydrophobic properties of the materials. The performed microbiological assays revealed that fibrous materials containing 5A8Q and their complexes exhibited good antibacterial and antifungal efficacy. Their activity was stronger against bacteria *S. aureus* than against bacteria *E. coli* and fungi *C. albicans*. Cell viability tests using MTT showed that the presence of 5A8Q and its complexes in the fibrous materials resulted in a significant decrease in the HeLa and MCF-7 cancer cell viability for the various times of cell incubation. Moreover, the observed cytotoxicity of the mats against cancer cells was greater than that against non-cancer HaCaT keratinocytes. All these properties make the novel materials potential candidates for the design of wound healing materials and as drug delivery systems for local therapy of cervical and breast cancer.

## 1. Introduction

Electrospinning has attracted significant attention as a cutting-edge and diverse method for the producing of materials of continuous fibers with tunable structures and properties [1,2,3]. In electrospinning, a high voltage electrical field is applied to a polymer solution or melt, resulting in the deposition of fibers with diameters varying from a few nanometers to the µm on the collector surface. The materials prepared by electrospinning exhibit important specific properties, such as an intrinsically high surface-to-volume ratio, high porosity, flexibility in functionalization, being lightweight, etc. These materials are suitable for application in pharmacy, medicine, cosmetics, etc. [1,2,3,4,5,6]. Diverse-in-nature drugs can be loaded in electrospun fibrous materials and their release profile can be influenced by careful selection of the morphology and composition of the fibers [7,8]. The important characteristics of electrospun materials can contribute to reducing the undesirable side effects of drugs and enhancing their therapeutic efficacy.

Natural polymers have been extensively explored as carriers of drugs and biologically active compounds, owing to their renewability and wide distribution. Carboxymethyl cellulose (CMC) is a derivative of the most widely used natural polymer cellulose and possesses carboxymethyl groups bound to a cellulose backbone [9,10]. CMC is a polysaccharide that exhibits a set of valuable properties, i.e., biodegradability, biocompatibility and low toxicity. Due to these properties, CMC has numerous applications in various fields, including biomedicine and pharmacy [9,11,12,13]. It has been shown that electrospinning of CMC on its own to prepare continuous fibers is difficult due to its polyelectrolyte nature [14]. The preparation of CMC-based fibrous materials is feasible only in the presence of an electrospinnable polymer [11,14,15]. Poly(vinyl alcohol) (PVA) is a non-ionogenic water-soluble polymer that is one of the most commonly used for a number of biomedical applications since it is a non-toxic, biocompatible, biodegradable polymer, has good chemical and thermal stability and can be easily crosslinked [16]. Electrospinning of PVA is easily feasible. PVA-based fibrous materials have been demonstrated to be promising in terms of applications in biomedicine [17,18].

8-Hydroxyquinoline (8Q) and its derivatives have been reported to exert several activities, e.g., antibacterial, antifungal, anticancer, antiviral and antioxidant [19,20]. The chelating ability of these compounds towards biologically significant transition metal ions (Cu^2+^, Fe^2+^, Fe^3+^, etc.) has an impact on their biological properties [19,20]. In our previous studies, 8Q derivatives have been loaded in fibrous mats obtained by electrospinning from synthetic polymers [21]. Materials containing 8Q have also been electrospun from mixed solutions of chitosan and poly(ethylene oxide) [22], of N-carboxyethyl chitosan and polyacrylamide [23], of cellulose acetate or cellulose acetate and poly(ethylene glycol) [24]. We have reported that these materials loaded with 8Q derivatives display good antibacterial and/or anticancer properties. Moreover, we have recently shown that electrospun fibrous materials composed of polylactide and a derivative of chitosan or polyetheramine and 8Q derivative and their complexes with Cu^2+^ (Fe^3+^) manifest high anticancer and/or antimicrobial activities [25,26].

Among the 8Q derivatives, 5-amino-8Q (5A8Q) was selected in this study because it is known to possess high antiproliferative activity towards a large number of human cancer cell lines [27,28]. 5A8Q also exhibits good antibacterial, antifungal and antioxidant activity [29]. We have demonstrated that, after modifying styrene/maleic anhydride copolymer mats with 5A8Q, they acquire antibacterial and antifungal activity [30]. In addition, 5A8Q has the ability to chelate metal ions [27].

The fabrication of fibrous materials from PVA, CMC and a model compound of the 8Q group 5A8Q has not yet been reported. It has been shown that the formation of complexes of 8Q derivatives with Cu and Fe ions—ions of biological importance—leads to an increase in the biological activity of these derivatives [20,31]. Therefore, in the present work, these ions have been chosen for the complex preparation.

The aim of the present study was to investigate the possibility of preparing novel antibacterial, antifungal and anticancer fibrous materials based on PVA, CMC and the model drug 5A8Q by electrospinning. Complexes between crosslinked PVA/CMC/5A8Q mats and Cu^2+^ and Fe^3+^ were obtained. The surface composition, thermal characteristics and morphology of the prepared materials were assessed in detail by ATR-FTIR spectroscopy, EDX spectroscopy, XPS spectroscopy, thermogravimetric analysis (TGA) and SEM. Considering the potential biomedical application, the activity of mats containing 5A8Q and their complexes against pathogenic microorganisms *S. aureus*, *E. coli* and *C. albicans* was estimated. The biological behavior of the obtained fibrous materials upon contact with human cancer cells such as HeLa and MCF-7, as well as non-cancer human keratinocytes such as HaCaT, was also studied.

## 2. Materials and Methods

### 2.1. Materials

Sodium carboxymethyl cellulose (CMC), with average Mw 250,000, degree of substitution 1.2 and 5-amino-8-hydroxyquinoline dihydrochloride (5A8Q), was obtained from Aldrich (St. Louis, MO, USA). Poly(vinyl alcohol) (PVA), with Mw 85,000–124,000 g/mol (96% hydrolyzed), CuCl_2_ anhydrous and FeCl_3_ anhydrous, was provided from Acros Organics (Geel, Belgium). Absolute ethanol, glutaraldehyde (50% *v*/*v*) and all salts utilized for the obtaining of buffer solutions (KH_2_PO_4_, Na_2_HPO_4_) were delivered from Merck Chemicals (Merck, Billerica, MA, USA). The buffer solution (KH_2_PO_4_/Na_2_HPO_4_) with a pH of 7.4 and an ionic strength of 0.1 was applied.

Human cervical adenocarcinoma (HeLa) and breast cancer (MCF-7) cell lines were purchased from the American Type Culture Collection (ATCC, Manassas, VA, USA). CLS Cell Lines Service (Eppelheim, Germany) delivered the human keratinocyte cell line HaCaT. Dulbecco’s modified eagle medium (DMEM) from Sigma-Aldrich, Schnelldorf, Germany, which was supplemented with 10% fetal bovine serum (FBS) from Gibco/BRL (Grand Island, NY, USA), penicillin (100 U/mL) and streptomycin (100 µg/mL) from LONZA, Cologne, Germany and 2 mM L-glutamine from LONZA (Cologne, Germany), was utilized for culturing of cells. Acridine orange (AO), ethidium bromide (EtBr) and 3-(4,5-Dimethylthiazol-2-yl)-2,5-diphenyltetrazolium bromide (MTT) were delivered from Sigma-Aldrich, Schnelldorf, Germany. 4′,6-diamidino-2-phenylindole (DAPI) supplied from AppliChem, Darmstadt, Germany was used. The Orange Scientific, Braine-l’Alleud, Belgium delivered the plastic disposable consumables. *S. aureus* strain 749, *E. coli* strain 3588 and *C. albicans* strain 74 were obtained from the National Bank for Industrial Microorganisms and Cell Cultures (NBIMCC, Sofia, Bulgaria).

### 2.2. Preparation of the PVA/CMC and PVA/CMC/5A8Q Solutions and Electrospinning

The homogenous CMC solution (1.5 wt%) was prepared by dissolving CMC in distilled water under stirring for 4 h. An aqueous PVA solution (11 wt%) was obtained by heating at 80 °C (12 h). The solutions consisting of PVA and CMC were obtained by mixing aqueous solutions of the polymers at PVA:CMC weight ratios of 9:1, 8:2 and 7:3. For the preparation of the PVA/CMC/5A8Q solution, CMC (0.06 g) was dissolved in distilled water (3 mL) and after the complete dissolution of CMC, 0.069 g 5A8Q was added (ratio of (5A8Q)/(CMC-units) was 1.18/1 (mol/mol)). The resulting solution was agitated for 1 h. To prepare the PVA/CMC/5A8Q mixed solution with 5A8Q content 5wt% (relative to the polymer weight) and PVA/CMC = 8:2 *w*/*w*, 12 g 11 wt% PVA aqueous solution was added to the obtained CMC/5A8Q solution and stirred for 30 min. The same procedure was used for obtaining the PVA/CMC/5A8Q mixed solution with 5A8Q content 3wt% (relative to the weight of polymers) and PVA/CMC = 8:2 *w*/*w* (ratio of (5A8Q)/(CMC-units) was 0.71/1 (mol/mol)).

The obtained mixed solutions were transferred in a needle-equipped syringe (gauge: 20GX11/2″) and were delivered with the use of an infusion pump (NE-300 Just InfusionTM Syringe Pump, New Era Pump Systems Inc., Farmingdale, NY, USA) at a rate of 0.7 mL per h. Electrospinning of PVA/CMC and PVA/CMC/5A8Q solutions was conducted at 18 kV applied voltage from a custom-made high voltage DC power supply, constant distance between the needle and the grounded collector of 11 cm, collector rotation speed of 1300 rpm, temperature 25 °C and ca. 40% relative humidity. The electrode of positive polarity was attached to the needle with the crocodile clip. A grounded rotating drum (diameter of 56 mm) was used as a collector. Aluminum foil was deposited on the collector to facilitate the fiber collection. The obtained fibrous materials were dried additionally under reduced pressure (0.01 mmHg) at 30 °C for 12 h. After electrospinning, PVA/CMC and PVA/CMC/5A8Q mats were crosslinked with glutaraldehyde vapors at 25 °C for 24 h. The crosslinked fibrous materials (designated as cr(PVA/CMC) and cr(PVA/CMC)/5A8Q) were dried under reduced pressure (0.01 mmHg) at 30 °C.

### 2.3. Obtaining the Cu^2+^ (Fe^3+^) Complexes of cr(PVA/CMC)/5A8Q Mats and of 5A8Q

cr(PVA/CMC)/5A8Q mats were placed in an ethanol solution of CuCl_2_ or FeCl_3_ (0.1M) for 50 min at 25 °C to produce their Cu^2+^ or Fe^3+^ complexes (designated as cr(PVA/CMC)/5A8Q,Cu^2+^ and cr(PVA/CMC)/5A8Q,Fe^3+^). Then, the mats were washed with ethanol to remove the adsorbed CuCl_2_ (FeCl_3_) and then freeze-dried.

The Cu^2+^ (Fe^3+^) complexes of 5A8Q (designated as 5A8Q,Cu^2+^ and 5A8Q,Fe^3+^) were synthesized using a procedure detailed in Supplementary Material (see synthesis of the 5A8Q,Cu^2+^ (Fe^3+^)).

### 2.4. Characterization

A Bookfield DV-II+ programmable viscometer produced by Middleboro, MA, USA, outfitted with a thermostatic cup for the sample and a cone spindle for the cone/plate option was used to determine the dynamic viscosity of the prepared spinning solutions at 25 ± 0.1 °C. The electrical resistance of the solutions was determined in an electrolytic cell outfitted with rectangular sheet platinum electrodes, as previously reported in detail [32].

The morphology of the prepared materials was examined by scanning electron microscopy (SEM) with the aid of a Jeol JSM-5510 (Tokyo, Japan) SEM microscope. The electrospun mats were sputtered with gold prior to SEM imaging. At least 30 fibers from three different SEM micrographs resulting in a total of 90 fibers per mat were measured to evaluate the morphology of fibers using the ImageJ software (V.1.53 e, Wayne Rasband, National Institute of Health, Bethesda, MD, USA). The elemental mapping of the vacuum-coated with carbon materials was carried out using Tescan Lyra (Brno, Czech Republic) SEM microscope equipped with Quantax (Bruker, Billerica, MA, USA) energy-dispersive X-ray spectrometer (EDX) fitted with XFlash 5010 (Bruker) detector.

The thicknesses of the fibrous materials were measured using a micrometer. The apparent density ρ_mat_ and porosity P_mat_ of the mats was calculated according to the equations:

(1) $$\rho_{\mathrm{mat}}=\frac{M_{\mathrm{mat}}}{d_{\mathrm{mat}}\times A_{\mathrm{mat}}}$$

where M_mat_ is mass of the mat, d_mat_ is thickness of the mat and A_mat_ is the area of the mat and

(2) $$P_{\mathrm{mat}}\;(\%)=\left(1-\frac{\rho_{\mathrm{mat}}}{\rho_{\mathrm{bulk}}}\right)\times 100$$

where ρ_bulk_ is the bulk density of PVA (1.19 g/cm^3^) or the blend of PVA/CMC (density of CMC is 1.6 g/cm^3^).

The surface chemical composition of the prepared materials was analyzed using XPS. The analyses were conducted in the ultrahigh vacuum (UHV) chamber of an ESCALAB MkII (VG Scientific, East Grinstead, UK) spectrometer with Mg Kα excitation. For energy calibration as a reference, the C1s line at 285 eV was utilized.

The water contact angles (WCA) were determined by the static sessile drop method. For this purpose, an Easy Drop Krüss GmbH apparatus (DSA 10-MK2 model, Hamburg, Germany) was used. A total of 10 μL of deionized water was dropped on the material surface. A photograph of each droplet on the mat was acquired (20 s after droplet deposition and was checked after 20 min). The WCA values were measured through computer analysis of the obtained photos of the droplets. The information collected comprised the means of 20 measurements for each sample.

Attenuated total reflection Fourier-transform infrared (ATR-FTIR) spectra were obtained by an IRAffinity-1 spectrophotometer produced by Shimadzu Co., Kyoto, Japan, fitted with a MIRacleTM ATR accessory with diamond crystal (PIKE Technologies, Madison, WI, USA) in the range of 4000–600 cm^−1^.

Thermogravimetric analysis (TGA) was conducted on a Perkin Elmer TGA 4000 (Waltham, MA, USA) at 10 °C/min heating rate in an argon atmosphere. For instrument control, data collecting and data processing, Pyris v.11.0.0.0449 software was utilized.

To determine the stability of cr(PVA/CMC)/5A8Q mats in PBS of pH 7.4, the mats were placed in the buffer for 24 h and 72 h. The treated samples were repeatedly washed with distilled water and then were kept in a freezer at −20 °C for 8 h. The pre-frozen mats were then quickly placed in a freeze drier (Martin Christ Alpha 1-2 LDplus, Osterode am Harz, Germany) and then freeze-dried above the ice condenser chamber; the morphology of fibers was observed using a Jeol JSM-5510 SEM microscope (Tokyo, Japan). The weight losses of cr(PVA/CMC)/5A8Q mats and their complexes were determined after the immersion of the mats in PBS for 24 h and 72 h and subsequently freeze-dried.

The swelling degree (α) of cr(PVA/CMC)/5A8Q mats and their complexes after 480 min and 24 h in PBS (pH 7.4) was determined gravimetrically. The degree was calculated by using the following equation:

α% = (W_sm_− W_dm_)/W_dm_ × 100 (3)

where W_sm_ is the weight of the swollen mat and W_dm_ is the weight of the dry mat.

### 2.5. In Vitro Release of 5A8Q (5A8Q,Cu^2+^ (Fe^3+^)) from the Fibrous Mats

The 5A8Q (5A8Q,Cu^2+^ (Fe^3+^)) release was investigated in vitro at 37 °C in PBS (pH 7.4) containing Tween 40 (PBS/Tween 40 was 99/1 *v*/*v*). The fibrous mats containing 5A8Q (5A8Q,Cu^2+^ (Fe^3+^)) (25 mg) were immersed in the 100 mL of PBS containing Tween 40 under stirring at 100 rpm. At predetermined time intervals, aliquots were taken from the solutions. The absorbance of the aliquots was registered using a DU 800 UV–vis spectrophotometer produced by Beckman Coulter, Brea, CA, USA, at a wavelength of 266 nm for mats containing 5A8Q, of 275 nm for mats containing 5A8Q,Cu^2+^ and of 278 nm for mats containing 5A8Q,Fe^3+^. The cumulative amount of 5A8Q (5A8Q,Cu^2+^ (Fe^3+^)) released from the fibrous materials was measured from the appropriated calibration curves. The determination at each time point was carried out in triplicate; the error bars on the graphs represent the standard deviation. The total amount of 5A8Q (5A8Q,Cu^2+^ (Fe^3+^)) in the mats was calculated by dissolving three samples (20 mg) in 100 mL of ethanol at 37 °C and determining the absorbance using the above-mentioned UV–vis spectrophotometer at a wavelength of 360 nm for mats containing 5A8Q, of 326 nm for mats containing 5A8Q,Cu^2+^ and of 350 nm for mats containing 5A8Q,Fe^3+^.

### 2.6. Evaluation of the Antibacterial and Antifungal Activity of the Fibrous Mats

The minimum inhibitory concentration (MIC) of 5A8Q and 5A8Q,Cu^2+^ (Fe^3+^) was estimated for *S. aureus* 749, *E. coli* 3588 and *C. albicans* 74, respectively (for estimation of the MIC see Supplementary Material).

The disk diffusion assay was used for assessment of the antibacterial and antifungal properties of the fibrous materials loaded with 5A8Q and their complexes towards the Gram-positive bacteria *S. aureus* and Gram-negative bacteria *E. coli* and towards the fungi *C. albicans*. All prepared fibrous materials were cut into discs with a diameter of 16 mm. The tryptic soy agar (TSA) solid medium obtained from Becton Dickinson, Heidelberg, Germany for bacteria *S.aureus* and *E.coli* and Sabouraud dextrose agar (SDA) solid medium delivered from Becton Dickinson, Sparks, MD, USA for fungi *C. albicans* were used in the studies. The petri dishes containing solid agar were inoculated with 0.1 mL of suspension of *S. aureus* (*E. coli*) cultured for 24 h or *C. albicans* cultured for 48 h (1 × 10^5^ cells/mL). The discs were positioned on the inoculated surface within 5 to 10 min after inoculation. The dishes with fibrous discs were positioned at 37 °C for 24 h for *S. aureus* (*E. coli*) and at 37 °C for 48 h for *C. albicans*. Finally, the zones of inhibition around each disk were measured. Using ImageJ software (V.1.53 e, Wayne Rasband, National Institute of Health, Bethesda, MD, USA), the mean values of the inhibition zones were calculated based on 15 measurements.

### 2.7. MTT Cell Viability Assay

Evaluation of the viability of normal and cancer human cell lines in the presence of fabricated fibrous mats was performed by the MTT test [33]. The cells were trypsinized with 0.25% Trypsin–EDTA and counted by a hemocytometer. The cells were seeded at a density of 1 × 10^5^ cells/mL in 96-well microtiter plates in DMEM medium and were cultivated overnight to form a monolayer. Thereafter, the fibrous materials (crPVA, cr(PVA/CMC), cr(PVA/CMC)/5A8Q, cr(PVA/CMC)/5A8Q,Cu^2+^ and cr(PVA/CMC)/5A8Q,Fe^3+^) that had been sterilized by UV light for 30 min were added to the wells (six replicates per sample), and incubated for two time intervals (24 h and 72 h) at 37 °C, 5% CO_2_ and 95% relative humidity**.**_._ All fibrous materials loaded with 5A8Q and their complexes contained 20 µM 5A8Q. Untreated cell cultures were used as controls. The cell lines were also exposed to the solutions of 5A8Q and its complexes. At the end of the exposure time, 100 μL of MTT solution (5 mg/mL in PBS) was added to each well and incubated for an additional 3 h. Finally, the supernatant was removed, the formed purple–blue formazan crystals were dissolved with DMSO:ethanol (1:1 (*v*/*v*)) solution and the sample absorbances (A) were measured at 570 nm by an ELISA reader produced by TECAN, Sunrise™, Grodig/Salzburg, Austria. The cell viability was proportional to the extent of formazan production. The absorbance values obtained from the wells of untreated cells, i.e., cells cultured only in DMEM medium, represented 100% cell viability.

The following equation was used to determine cell viability:

Cell viability (%) = A_570_ (experimental)/A_570_ (control) × 100 (4)

### 2.8. Detection of Cell Morphological Alterations by Fluorescence Microscopy

#### 2.8.1. Acridine Orange/Ethidium Bromide Live–Death Staining

To observe morphological changes, control and treated cervical and mammary carcinoma cell cultures and normal human HaCaT keratinocyte cells were stained with AO and EtBr according to standard procedure [34]. The cells (1 × 10^5^ cells/well) were grown overnight on glass slides (placed on the bottom of a 24-well tissue culture plate) in DMEM growth medium at 37 °C and 5% CO_2_. After that, the fibrous materials (crPVA, cr(PVA/CMC), cr(PVA/CMC)/5A8Q, cr(PVA/CMC)/5A8Q,Cu^2+^ and cr(PVA/CMC)/5A8Q,Fe^3+^) and solutions of 5A8Q and its complexes were placed in the subconfluent cell cultures and further incubated for 24 h. The glass slides were then rinsed with PBS and stained with AO/EtBr solution (5 μg/mL AO, 5 μg/mL EtBr; *v*:*v* = 1:1). Images were captured instantly with a Leica DM 5000B fluorescence microscope (Wetzlar, Germany).

#### 2.8.2. DAPI Staining

Cells were cultured and treated as described in paragraph 2.8.1. After washing the glass slides with PBS, the cells were fixated with 3% paraformaldehyde at 25 °C. Then, staining for 15 min in the dark with a DAPI solution was carried out. A Leica DM 5000B fluorescence microscope (Wetzlar, Germany) was used to investigate the morphology of the nuclei of the treated and untreated control cells.

### 2.9. Statistical Analysis

All data are presented as mean ± standard deviation (SD). The statistical significance of the differences between the relative cell viabilities of control and treated cell cultures was evaluated by one-way analysis of variance (ANOVA), followed by the Bonferroni’s post hoc test (GraphPad PRISM Software Inc., version 5, San Diego, CA, USA). Values of *p* < 0.05 were accepted as statistically significant.

## 3. Results and Discussion

### 3.1. Morphology

**Figure 1:**
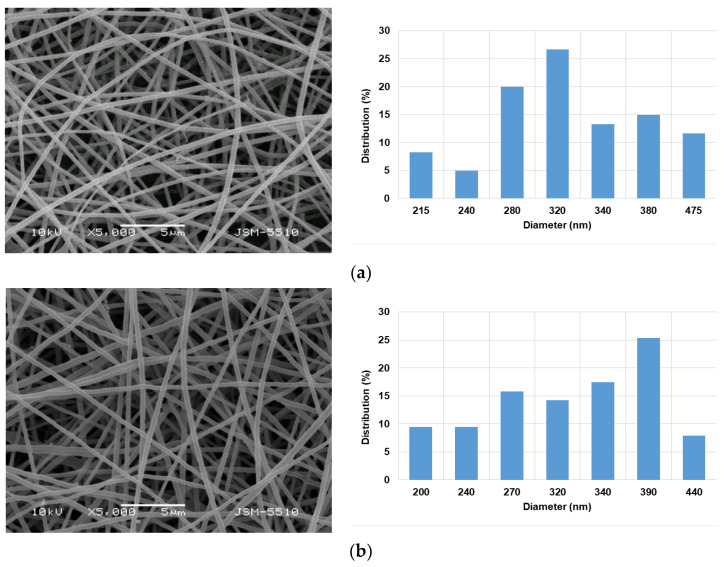
SEM images and fiber diameter distribution of fibrous materials of: (**a**) PVA/CMC/5A8Q (3 wt% 5A8Q); (**b**) PVA/CMC/5A8Q (5 wt% 5A8Q); magnification ×5000.

It has been shown that the addition of the non-ionogenic polymer PVA to the spinning solution can assist the electrospinning of the ionogenic polymer CMC [15,35,36]. Therefore, PVA was chosen as a partner in the preparation of fibrous materials containing CMC. Preliminary experiments were conducted to choose such a PVA/CMC weight ratio of the spinning solutions to incorporate the model drug 5A8Q that produced defect-free and cylindrical fibers. As seen in Figure S1a–d (Supplementary Material), fibrous materials with different morphologies were obtained by altering the ratio of PVA/CMC. At the weight ratio of PVA/CMC = 8:2, cylindrically shaped continuous fibers without defects having a mean diameter of 340 ± 80 nm were prepared. With the increase of the CMC content (PVA/CMC = 7:3 *w*/*w*), the mean fiber diameter decreased (225 ± 47 nm) (Supplementary Material, Figure S1d). In the case of PVA/CMC = 7:3 *w*/*w*, a small number of spindle-shaped defects were also detected. The observed decrease in fiber diameter and the appearance of defects was most likely due to the increase in conductivity of the solutions with increasing CMC content (Supplementary Material, Table S1). Therefore, in the present study, spinning solutions with a PVA/CMC ratio = 8:2 *w*/*w* were chosen for the preparation of PVA/CMC fibrous mats with incorporated 5A8Q. The presence of an ionized carboxylic group in CMC may permit electrostatic interaction between the CMC molecules of the mats containing CMC and the 5A8Q. The molar ratio between 5A8Q and CMC units was close to 1/1—1.18/1 and 0.71/1 for the spinning solutions, with 5 wt% and 3 wt% 5A8Q content relative to the polymer weight, respectively. The SEM micrographs and the distribution of the fiber diameters of the obtained PVA/CMC/5A8Q mats are presented in Figure 1a,b. As can be seen from the SEM images, the fabricated fibers were cylindrical, continuous and without defects. The mean diameters were 330 ± 80 nm and 324 ± 80 nm for the PVA/CMC fibrous materials loaded with 3 wt% and 5 wt% 5A8Q, respectively. These diameters were close to those of the PVA/CMC mats. This fact might be explained by the slight change in the solution conductivity upon addition of 5A8Q to the PVA/CMC solution (Supplementary Material, Table S1). The PVA/CMC solution viscosity was slightly reduced when 5A8Q was added to the solution (Supplementary Material, Table S1).

A porosity of 64.0 ± 1.2% was determined for the PVA/CMC (8:2 *w*/*w*) fibrous materials. The porosity values of the PVA/CMC fibrous materials containing 3 wt% and 5 wt% 5A8Q were 59.0 ± 2.0% and 56.0 ± 1.5%, respectively. The difference between the determined values was not considered statistically significant.

**Figure 2:**
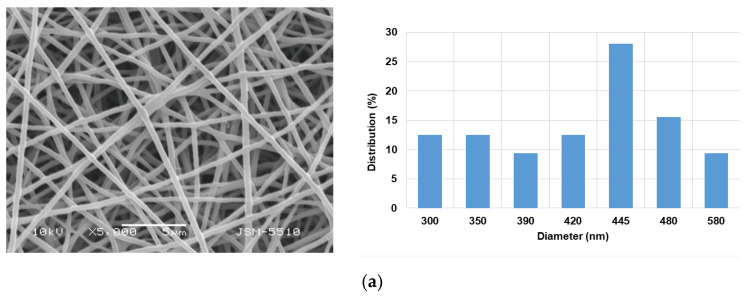

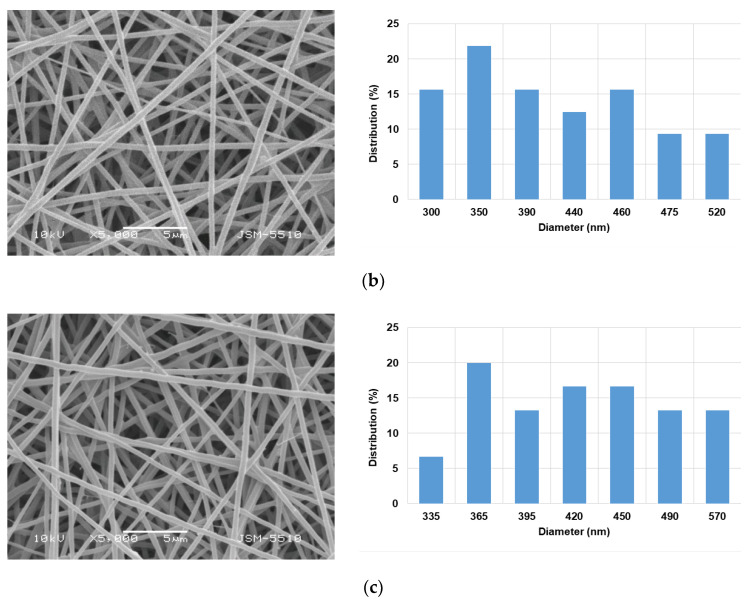
SEM images and fiber diameter distribution of fibrous materials of: (**a**) cr(PVA/CMC)/5A8Q (5 wt% 5A8Q) after immersion in PBS (pH 7.4) for 24h; (**b**) cr(PVA/CMC)/5A8Q (5 wt% 5A8Q) after treatment with a solution of CuCl_2_ and (**c**) FeCl_3_ for 50 min.

The water solubility of PVA/CMC/5A8Q fibrous materials might limit their potential application in medicine. To render the fibers insoluble, crosslinking with glutaraldehyde was carried out. The stability of the crosslinked PVA/CMC/5A8Q fibrous mats in aqueous medium was assessed after immersing the mats for 24 h in PBS. As can be seen from Figure 2a, the structure of these mats remained fibrous.

Swelling of the mats was observed, with the average diameter of the crosslinked PVA/CMC/5A8Q mats increasing to 425 ± 80 nm. Weight losses of 4 wt% and 4.6 wt% were determined for the crosslinked mats containing 5A8Q when immersed in PBS for 24h and 72h, respectively, and subsequently freeze-dried. These losses were close to the amount of 5A8Q loaded in the mats.

Complexes between the cr(PVA/CMC)/5A8Q fibrous materials and Cu^2+^ and Fe^3+^ ions were formed by a one-step treatment of the materials with a solution of CuCl_2_ and FeCl_3_ in ethanol for 50 min. It was found that under this treatment, the fibrous structure of the mats was preserved (Figure 2b,c). The mean fiber diameters increased slightly by up to 407 ± 70 nm and up to 435 ± 70 nm for Cu^2+^ and Fe^3+^ complexes of cr(PVA/CMC)/5A8Q, respectively. The stability of the complexes of crosslinked mats containing 5A8Q after immersion in PBS for 24h and 72h was also determined. The measured weight losses were close to the amount of 5A8Q,Cu^2+^ (Fe^3+^) incorporated in the mats. The registered losses were 3.6 wt% and 4.3 wt% for the cr(PVA/CMC)/5A8Q,Cu^2+^ mat and 3.5 wt% and 4.1 wt% for the Fe^3+^ complex of the mat, respectively.

The elemental mapping performed by EDX of cr(PVA/CMC)/5A8Q fibers demonstrated the presence of expected C, O, N, Na and Cl (Supplementary Material, Figure S2). In the case of cr(PVA/CMC)/5A8Q,Cu^2+^ fibrous materials, the elemental mapping revealed the presence of a peak for Cu in addition to the peaks characteristic for C, O, N, Na and Cl (Supplementary Material, Figure S3). The EDX analyses of cr(PVA/CMC)/5A8Q,Fe^3+^ fibers showed the appearance of a peak for Fe (Supplementary Material, Figure S4). In this case, the peaks for C, O, N, Na and Cl were also detected. These results confirmed the formation of Cu^2+^ or Fe^3+^ complexes of the mats containing 5A8Q.

### 3.2. ATR-FTIR Spectra of the Mats

**Figure 3:**
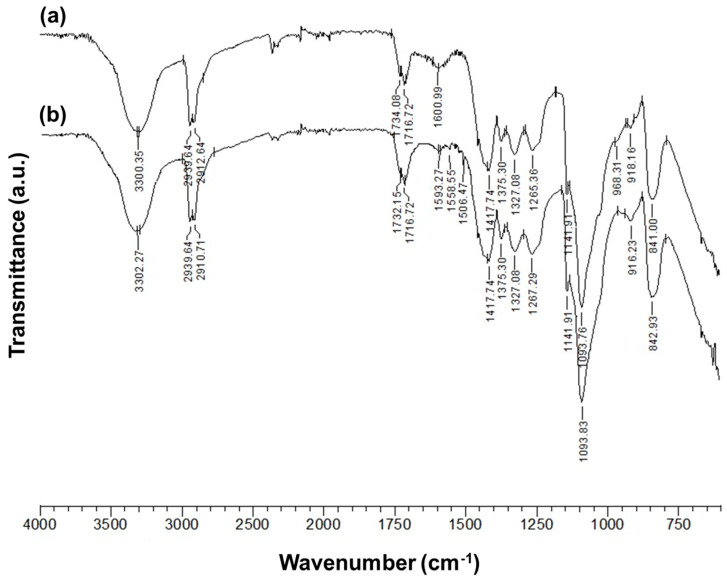

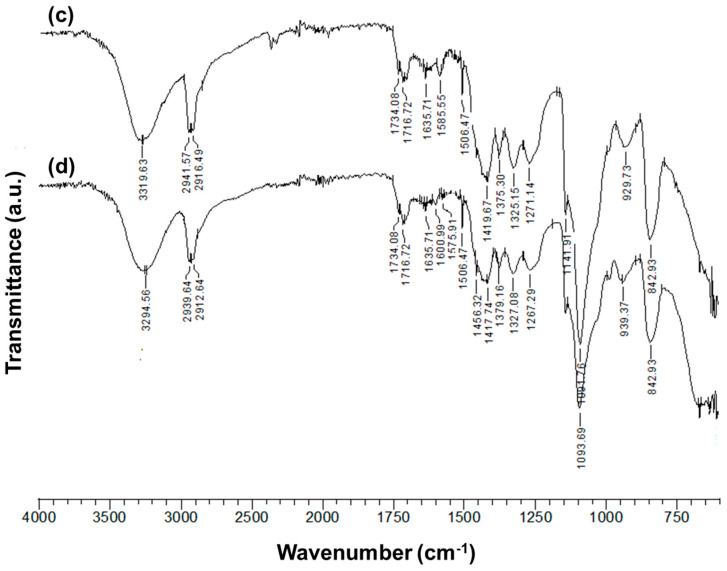
ATR-FTIR spectra of fibrous materials from: (**a**) cr(PVA/CMC); (**b**) cr(PVA/CMC)/5A8Q; (**c**) cr(PVA/CMC)/5A8Q,Cu^2+^; (**d**) cr(PVA/CMC)/5A8Q,Fe^3+^.

The cr(PVA/CMC)/5A8Q fibrous materials and their complexes were analyzed using ATR-FTIR spectroscopy (Figure 3). The spectrum of the cr(PVA/CMC) mat (Figure 3a) showed characteristic bands at 1601 cm^−1^, 1418 cm^−1^ and 1327 cm^−1^, respectively, for ν_as_ and ν_s_ of COO^-^ in the carboxymethyl groups (R-CH_2_OCOO^-^) of CMC and in-plane δ of -C-CH and O-CH- in the carboxymethyl groups of CMC [36], with the exception of the bands of PVA (3300 cm^−1^ assigned to ν_O-H_ of PVA and CMC; 2940 and 2913 cm^−1^ assigned to aliphatic ν_C-H_; 1734 cm^−1^ and 1717 cm^−1^ assigned to ν_C = O_ from residual vinyl acetate repeating units of PVA). As Figure 3b shows, in the case of the ATR-FTIR spectrum of the cr(PVA/CMC)/5A8Q mat, the presence of 5A8Q led to a bathochromic shift of the band for ν_as_ of COO^-^ of CMC by 7 cm^−1^ to 1593 cm^−1^. This feature demonstrated that interactions between CMC and 5A8Q occurred. New bands at 1558 and 1506 cm^−1^, attributed to the stretching vibrations of the 5A8Q ring, also appeared.

The spectra of cr(PVA/CMC) and cr(PVA/CMC)/5A8Q mats showed (Figure 3a,b) that the C-O-C stretching band of PVA shifted (4 cm^−1^) to a higher wavenumber compared with non-crosslinked mats (Supplementary Material, Figure S5b,c). According to the literature [37], this was attributed to C-O-C bonds from acetal and ether groups formed during the crosslinking of PVA with glutaraldehyde. A drop in the ratio of the intensity of the bands at 3300 cm^−1^ and at 2940 cm^−1^ was also detected compared with the spectra of non-crosslinked fibrous materials, revealing the consumption of OH groups as a result of the crosslinking of PVA and CMC (Supplementary Material, Figure S5b,c). These observations proved that crosslinking of PVA and CMC proceeded successfully. The bands related to ν_C = N_ from 5A8Q (1568 cm^−1^) (Supplementary Material, Figure S5a) in the ATR-FTIR spectra of the complexes with Cu^2+^ and Fe^3+^ (Figure 3c,d) were shifted to the higher wavenumbers by 18 cm^−1^ to 1586 cm^−1^ for the cr(PVA/CMC)/5A8Q,Cu^2+^ mat and by 8 cm^−1^ to 1576 cm^−1^ for the cr(PVA/CMC)/5A8Q,Fe^3+^ mat, respectively. The observed shifts were probably attributed to the fact that the lone pair on the nitrogen of 8Q ring from 5A8Q took part in the bonding with the Cu and Fe ion.

### 3.3. Water Contact Angle Measurements

**Figure 4:**
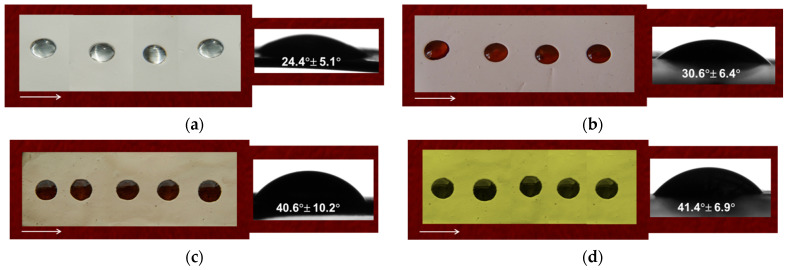
Photographs of water droplets placed on the surfaces of mats from (**a**) cr(PVA/CMC); (**b**) cr(PVA/CMC)/5A8Q; (**c**) cr(PVA/CMC)/5A8Q,Cu^2+^; (**d**) cr(PVA/CMC)/5A8Q,Fe^3+^. An arrow points in the direction of the collector’s rotation.

The adhesion of bacterial, fungal and cancer cells and their proliferation can be strongly influenced by the hydrophilic/hydrophobic properties of fibrous mats [38]. Therefore, measurement of the contact angle of the fabricated materials was performed. Figure 4 displays digital images of the water droplets that were placed on the fibrous materials’ surfaces. It was found that the cr(PVA/CMC) fibrous materials were hydrophilic (Figure 4a). The registered WCA was 24.4 ± 5.1°. The incorporation of 5A8Q in the fibers slightly altered the hydrophilic/hydrophobic behavior of the cr(PVA/CMC) mats (the WCA value was 30.6 ± 6.4°, Figure 4b). The complex formation between Cu^2+^ (Fe^3+^) and the cr(PVA/CMC)/5A8Q mats resulted in a slight increase in the WCA values of the prepared materials (Figure 4c,d). The prepared Cu^2+^ (WCA: 40.6 ± 10.2°) and Fe^3+^ complexes (WCA: 41.4 ± 6.9°) of the mats remained hydrophilic.

### 3.4. Thermal Analysis of the Mats

**Figure 5:**
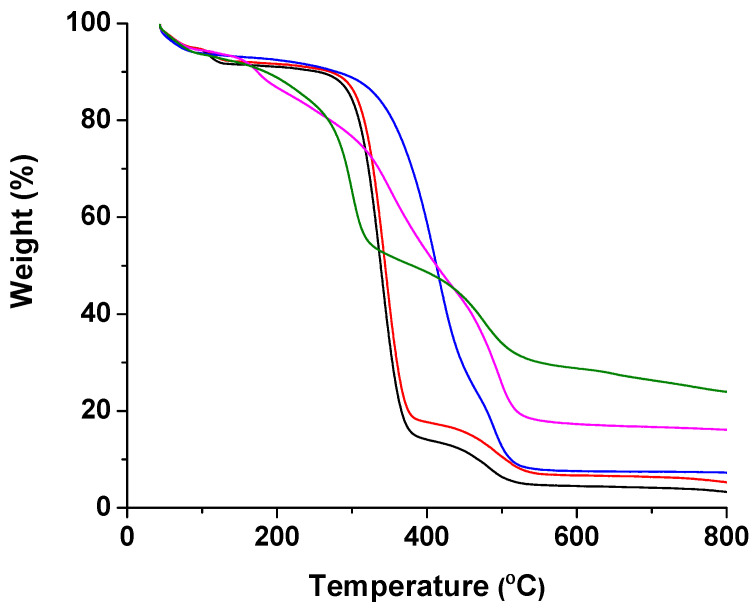
TGA thermograms of crPVA mat (black color), cr(PVA/CMC) mat (red color), cr(PVA/CMC)/5A8Q mat (blue color), cr(PVA/CMC)/5A8Q,Cu^2+^ mat (magenta color) and cr(PVA/CMC)/5A8Q,Fe^3+^ mat (olive color) in the range 50 to 800 °C.

The thermal stability of the cr(PVA/CMC)/5A8Q mats and their complexes was estimated by TGA. The thermograms of the obtained materials are presented in Figure 5. The crPVA mat degraded thermally in three stages. The first stage (45–130 °C) corresponded to the evaporation of the absorbed moisture, the second stage of degradation occurred in the temperature range 170–410 °C (T_max_ 338 °C) and was mainly due to the degradation of PVA side groups, while the third stage was in the temperature range 410–600 °C (T_max_ 477 °C) and was due to the degradation of the main chain of PVA [39].

Similar to the crPVA mat, the cr(PVA/CMC) mat degraded in three stages. The presence of CMC in the crPVA/CMC mat did not significantly affect the maximum degradation temperatures, which were 345 °C and 512 °C for the second and third degradation stages, respectively. The second stage of degradation, characterized by the greatest weight loss, started at 150 °C and ended at 438 °C and was due to the degradation of the PVA side groups, the decarboxylation of carboxyl groups in CMC, and the loss of CO_2_ [40]. For the cr(PVA/CMC)/5A8Q mat, the presence of 5A8Q led to a shift of the maximum degradation temperature for the second stage, characterized by the greatest weight loss, to a higher value of 416 °C; for the third stage it was 492 °C. The cr(PVA/CMC)/5A8Q,Cu^2+^ mat was degraded in four steps. The first stage of degradation was in the temperature range 45–100 °C with a weight loss of 4.5% due to desorption of ethanol or water; the second weight loss between 100 and 220 °C was 9.5%; the third weight loss between 220 and 424 °C was 38.5%; the fourth loss (424–600 °C) was 29.1% and was due to the decomposition of the cr(PVA/CMC)/5A8Q,Cu^2+^ mat. The TGA thermogram of the cr(PVA/CMC)/5A8Q,Fe^3+^ mat showed three stages of decomposition. The initial weight loss (5.7%) may have been due to the solvent molecules or moisture trapped inside the complexes. The maximum weight loss (41.2%) was observed in the temperature range of 100–375 °C; a weight loss of 23.3% was observed in the temperature range of 375–600 °C that was related to degradation of the cr(PVA/CMC)/5A8Q,Fe^3+^ mat. These results indicated that the maximum degradation temperatures of the cr(PVA/CMC)/5A8Q,Cu^2+^ (Fe^3+^) mat for the stage characterized by the greatest weight loss were shifted to lower temperatures (352 °C and 300 °C for the Cu^2+^ and Fe^3+^ complexes of the fibrous materials, respectively) compared with the maximum degradation temperature for the stage characterized by the greatest weight loss detected in the thermogram of the cr(PVA/CMC)/5A8Q mat (416 °C). This indicated that the coordination of Cu^2+^ or Fe^3+^ with the cr(PVA/CMC)/5A8Q mat led to a decrease in the thermal stability of the mat. The residual weight at 800 °C was 16.1% for the cr(PVA/CMC)/5A8Q,Cu^2+^ mat and 24% for the cr(PVA/CMC)/5A8Q,Fe^3+^ mat, respectively, corresponding to a mixture of metal oxide and some ash. From the TGA analyses, it was revealed that the metal oxide content in the residues was higher than calculated based on the complex formation between Cu^2+^ or Fe^3+^ with two molecules of 5A8Q incorporated in cr(PVA/CMC)/5A8Q mats. Therefore, it can be assumed that Cu^2+^ or Fe^3+^ most probably coordinated via O atoms of the PVA and CMC components of the mats, as well.

### 3.5. XPS Analysis of the Mats

XPS was used to study the surface composition of cr(PVA/CMC)/5A8Q mats and their complexes. The high resolution spectra of C_1s_, O_1s_, N_1s_, Na_1s_ and Cl_2p_ detected for cr(PVA/CMC)/5A8Q mats by XPS are presented in Figure S6a–e (Supplementary Material). Four peaks were recorded in the C_1s_ spectrum at 285.0 eV (-C-H or -C-C- from PVA, CMC and 5A8Q), 286.4 eV (-C-O or -C-OH from PVA and CMC; -C-OH and -C-N from 5A8Q), 289.0 eV (-O-C=O from PVA, -O-C-O- and -O-C=O from CMC and -O-C-O- from the acetal groups of crosslinked PVA) and 290.9 eV (π→π∗ shake-up satellite, characteristic for the 5A8Q ring) (Supplementary Material, Figure S6a). Regarding the O_1s_ spectrum, three peaks were observed: for oxygen atoms from -O-C-O- of CMC and from acetal groups of crPVA at 533.4 eV and for -C-O or -C-OH from PVA and CMC and -C-OH from 5A8Q at 532.6 eV, respectively, and a peak at 531.8 eV for -O-C=O from PVA and -O-C=O from CMC (Supplementary Material, Figure S6b). One peak, having two components, at 400.6 eV characteristic of -N-C from 5A8Q and at 401.6 eV assigned to the protonated amino group (-NH_3_^+^) from 5A8Q, was detected in the N_1s_ spectrum (Supplementary Material, Figure S6c). The spectrum of the cr(PVA/CMC)/5A8Q mat indicated the presence of a Cl_2p_ peak at 197.8 eV (Cl_2p3/2_) and at 199.4 eV (Cl_2p1/2_) from 5A8Q and of a Na_1s_ peak at 1071.4 eV from CMC (Supplementary Material, Figure S6d,e).

The formation of the Cu^2+^ and Fe^3+^ complexes on the surface of the cr(PVA/CMC)/5A8Q mats led to changes in the C_1s_, O_1s_ and N_1s_ spectra of the mats. In contrast to the cr(PVA/CMC)/5A8Q fibrous materials in the C_1s_ spectra of the complexes of the cr(PVA/CMC)/5A8Q mats, the appearance of a new peak at 287.0 eV assigned to -C-O---Cu (Fe) and -C-N---Cu (Fe) was detected (Supplementary Material, Figure S7a,g). A decrease in the area of the peak at 286.4 eV corresponding to C atoms from -C-O or -C-OH from PVA and CMC as well as from -C-OH and -C-N from 5A8Q was recorded. A new peak at 530.9 eV assigned to -O---Cu (Fe) appeared in the O_1s_ spectra of the complexes (Supplementary Material, Figure S7b,h). The N_1s_ spectra displayed the appearance of a new component at 399.7 eV ascribed to N atoms from 5A8Q coordinated to Cu^2+^ (Fe^3+^) (Supplementary Material, Figure S7c,i). In the Cu_2p_ spectrum of the cr(PVA/CMC)/5A8Q,Cu^2+^ mats, a peak composed of two components—Cu_2p1/2_ and Cu_2p3/2_—was recorded. Cu_2p3/2_ contained a major peak at 932.8 eV and two satellite peaks at 941.4 eV and 944.0 eV (Supplementary Material, Figure S7d). The major peak had binding energy that was close to that of Cu^2+^ complexes recorded by other authors (933.1 eV) [41]. This peak provided additional evidence for the successful complex formation between Cu^2+^ and the cr(PVA/CMC)/5A8Q mats. The new signal with two components Fe_2p1/2_ and Fe_2p3/2_ was registered in the Fe_2p_ region (Supplementary Material, Figure S7j). Each of the components consisted of a major peak and a satellite peak; for the Fe_2p1/2_ component, the peaks were recorded at 724.2 and at 730.4 eV, respectively. For the Fe_2p3/2_ component, the major peak was observed at 711.0 eV and the satellite at 715.0 eV. These results were consistent with the results reported by other authors for Fe^3+^ complexes [42] and additionally confirmed the Fe^3+^ complex formation in the surface layer of the cr(PVA/CMC)/5A8Q fibrous sample. As expected, for complexes of the cr(PVA/CMC)/5A8Q mats, a peak in the Cl_2p_ region (at 198.6 eV (Cl_2p3/2_) and 200.1 eV (Cl_2p1/2_)) (Supplementary Material, Figure S7e,k) was detected which confirmed the presence of Cl ions on the surface of the complexes.

### 3.6. In Vitro 5A8Q and Its Complexes’ Release Studies

**Figure 6:**
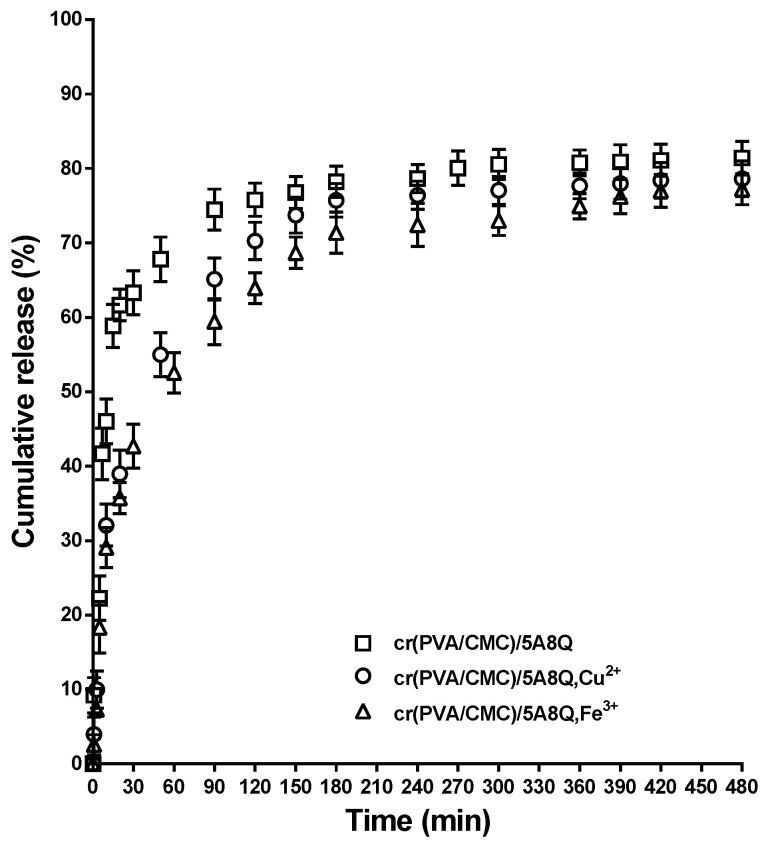
In vitro study of the 5A8Q, 5A8Q,Cu^2+^ and 5A8Q,Fe^3+^ release from mats: cr(PVA/CMC)/5A8Q (5 wt% 5A8Q), cr(PVA/CMC)/5A8Q,Cu^2+^ (5 wt% 5A8Q) and cr(PVA/CMC)/5A8Q,Fe^3+^ (5 wt% 5A8Q); PBS/Tween 80 (99/1 *v*/*v*), 37 °C, pH 7.4.

The in vitro release studies were carried out at 37 °C using a PBS buffer (pH 7.4) containing Tween 80 (99/1 *v*/*v*). The release profiles of 5A8Q and 5A8Q,Cu^2+^ (Fe^3+^) from cr(PVA/CMC)/5A8Q mats and their complexes are presented in Figure 6. A first stage of burst release was detected for all fibrous materials. Then, a second gradual mode of the release followed. It was found that cr(PVA/CMC) mats containing 5A8Q released ca. 54.4% of 5A8Q in the first 20 min. Around 40.0% and 37.7% of 5A8Q,Cu^2+^ and 5A8Q,Fe^3+^ were released from the respective fibrous materials during the initial 20 min, respectively. The amount of 5A8Q released from the cr(PVA/CMC)/5A8Q mat within 480 min was ca. 81.4%. The amount of 5A8Q,Cu^2+^ and 5A8Q,Fe^3+^ released from fibrous materials containing 5A8Q,Cu^2+^ and 5A8Q,Fe^3+^ was around 78.6 and 77.2% in 480 min, respectively. The amount of released 5A8Q and 5A8Q,Cu^2+^ (Fe^3+^) from the respective mats for 24 h did not deviate significantly from that detected for 480 min. The observed slight difference in the amount of 5A8Q and 5A8Q,Cu^2+^ (Fe^3+^) released from mats containing 5A8Q and 5A8Q,Cu^2+^ (Fe^3+^) was most likely due to the slight difference in the hydrophilic–hydrophobic characteristics of the mats. A WCA of 30.6 ± 6.4° was measured for the cr(PVA/CMC)/5A8Q fibrous materials, whereas the WCA values of the Cu^2+^ and Fe^3+^ complexes of cr(PVA/CMC)/5A8Q fibrous materials were 40.6 ± 10.2° and 41.4 ± 6.9°, respectively. The swelling degree, measured in a PBS buffer of pH 7.4 at 37 °C after 480 min and 24 h, for the cr(PVA/CMC)/5A8Q mat was 215.4 ± 3.0% and 216.5 ± 2.1%, for the cr(PVA/CMC)/5A8Q,Cu^2+^ mat was 157.9 ± 4.3% and 158.8 ± 3.7% and for the cr(PVA/CMC)/5A8Q,Fe^3+^ mat was 151.8 ± 2.8% and 152.5 ± 3.0%, respectively. All studied fibrous materials were hydrophilic and water swellable, which was favorable to water penetration and thus facilitated the 5A8Q and 5A8Q,Cu^2+^ (Fe^3+^) release.

### 3.7. Assessment of the Antibacterial and Antifungal Activities of the Mats

**Figure 7:**
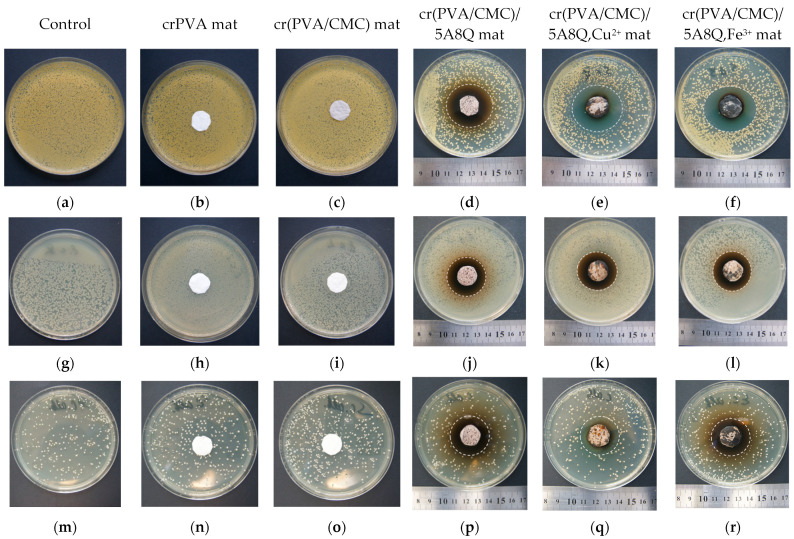
Images of the Petri dishes and inhibition zones towards *S. aureus* (**a**–**f**), *E. coli* (**g**–**l**) and *C. albicans* (**m**–**r**) observed around the fibrous materials.

The antibacterial and antifungal properties of cr(PVA/CMC)/5A8Q and their complexes against Gram-positive bacteria *S. aureus*, Gram-negative bacteria *E. coli* and fungi *C. albicans* were tested by disk diffusion assay. MIC values for 5A8Q and 5A8Q,Cu^2+^ (Fe^3+^) against the studied bacteria and fungi were also determined. It was found that 5A8Q and its complexes exhibited a stronger inhibitory activity towards Gram-positive bacteria *S. aureus* (MIC = 62.5 µg per mL) compared with towards Gram-negative bacteria *E. coli* (MIC = 250 µg per mL) and fungi *C. albicans* (MIC = 250 µg per mL). In Figure 7, the images of Petri dishes in which disks of the mats were put into contact with the studied bacterial or fungal cells are presented. For comparison, the antibacterial and antifungal activities of neat crPVA and cr(PVA/CMC) mats were also estimated. As seen in Figure 7b,c,h,i,n,o, these fibrous materials exerted no impact on the growth of pathogenic microorganisms and no inhibition was detected. The tests showed that the incorporation of 5A8Q in the materials and complexation with Cu^2+^ and Fe^3+^ resulted in the appearance of well-distinguished sterile zones around the fibrous disks, which was evidence of the presence of antibacterial and antifungal efficacy of the novel materials. In addition, fibrous materials containing 5A8Q and their complexes induced more pronounced suppression of the growth of bacteria *S. aureus* compared with those of bacteria *E. coli* and fungi *C. albicans*. In the microbiological screening against *S. aureus*, the measured mean diameters of the inhibition zones around disks of cr(PVA/CMC)/5A8Q fibrous materials and their Cu^2+^ and Fe^3+^ complexes were 38.0 ± 1.3, 39.0 ± 1.4 and 39.5 ± 2.4 mm, respectively (Figure 7d–f). The mean diameter values of the inhibition zones for the fibrous materials loaded with 5A8Q and their Cu^2+^ and Fe^3+^ complexes were 32.9 ± 0.2, 32.9 ± 0.9 and 33.1 ± 0.4 mm in the *E. coli* tests (Figure 7j–l) and 33.8 ± 0.5, 32.4 ± 0.5 and 32.7 ± 0.3 mm in the *C. albicans* tests (Figure 7p–r), respectively.

The findings of the performed investigations revealed that, in the case of cr(PVA/CMC)/5A8Q fibrous materials and their complexes, well-distinguished inhibition zones were detected. This finding implied that the incorporated 5A8Q and its complexes retained their antibacterial and antifungal efficacy. Still not very understood is the mode of action of 8Q derivatives in cells of bacteria and fungi. It is known that the efficacy of these compounds in inhibiting bacteria growth is dependent on their capability to chelate transition metal ions of biological importance [19]. 8Q derivatives can bind metallic prosthetic groups of bacterial enzymes, thus resulting in the disruption of the activity of the enzymes [43,44]. It has been suggested that the antibacterial effect of metal complexes of 8Q is due to their ability to block enzyme metal-binding sites of bacteria [44,45]. The antifungal properties of 8Q derivatives are based mainly on the damage of the cell membrane, resulting in cell death [46].

### 3.8. Evaluation of the Cytotoxicity of the Fibrous Mats towards HeLa and MCF-7 Cells and HaCaT Keratinocytes by MTT Assay

**Figure 8:**
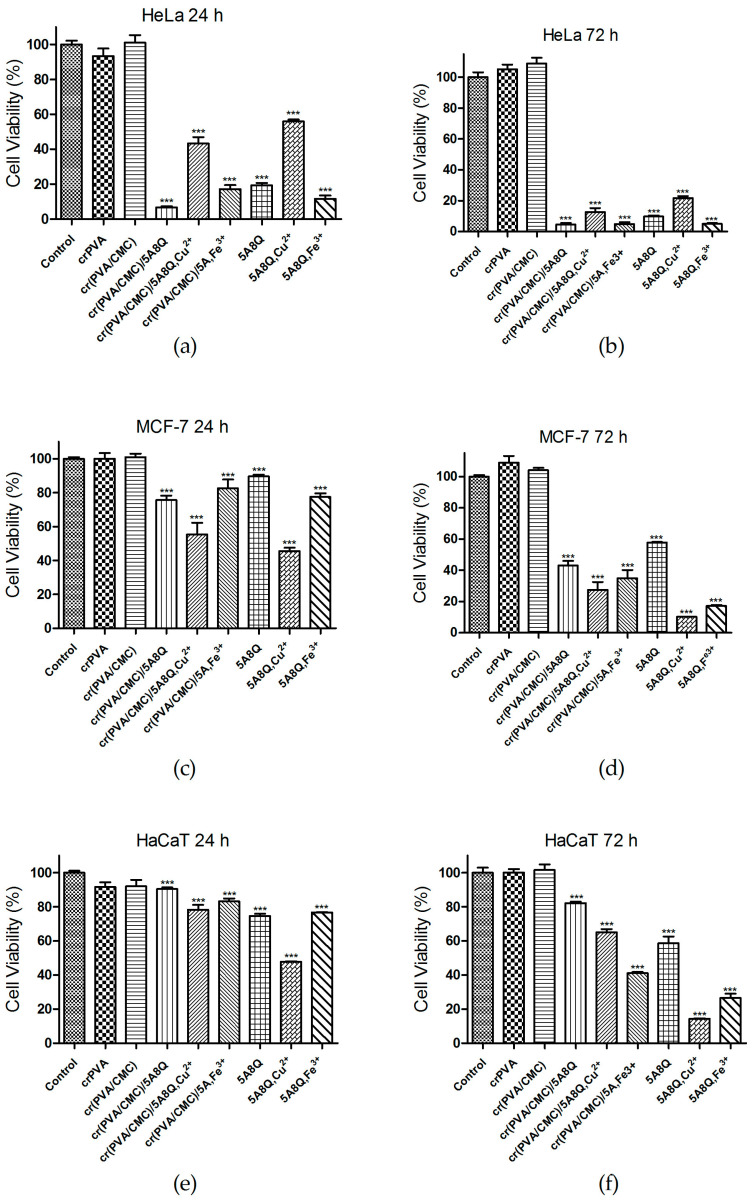
Viability of HeLa (**a**,**b**), MCF-7 (**c**,**d**) or HaCaT (**e**,**f**) cells after 24 (**a**,**c**,**e**) and 72 h (**b**,**d**,**f**) of incubation with different formulations. All formulations containing 5A8Q and complexes were studied at a concentration of 5A8Q 20 µM. Data are presented as mean ± SD; *** *p* < 0.001.

The anticancer activity of the prepared fibrous materials containing 5A8Q and their complexes was estimated using the MTT proliferation assay on the human carcinoma cell lines HeLa and MCF-7. The cytotoxicity of these mats towards normal HaCaT human keratinocyte cells was also evaluated. As shown in Figure 8, crPVA and cr(PVA/CMC) mats did not show any antiproliferative effect on all cell lines tested. The fibrous mats containing 5A8Q and their complexes induced a time-dependent reduction in the viability of HeLa cells (Figure 8a,b). The observed cytotoxic effect of the tested fibrous materials increased after 72 h of treatment. The cr(PVA/CMC)/5A8Q mats (4.5 ± 1.9% viability) and their Fe^3+^ complexes (4.8 ± 2.8% viability) displayed slightly stronger cytotoxicity towards Hela cells compared with the cr(PVA/CMC)/5A8Q,Cu^2+^ mats (12.6 ± 6.3% viability) at the 72nd hour. The inhibitory effect of 5A8Q-loaded mats and their complexes on HeLa cells was close to that found for free 5A8Q and its complexes (Figure 8b). As seen, the exposure of MCF-7 cells to cr(PVA/CMC)/5A8Q mats and their complexes caused a statistically significant decrease in cell viability that was more pronounced after 72 h of incubation (Figure 8c,d). The observed decline in the MCF-7 cell viability was less distinctive compared with that of HeLa cells. At the 72nd hour a more significant inhibition of MCF-7 cell viability was detected after treatment with the cr(PVA/CMC)/5A8Q,Cu^2+^ mats (16.7 ± 1.8% viability) than that induced by cr(PVA/CMC)/5A8Q mats (43.0 ± 7.3% viability) and their Fe^3+^ complexes (33.2 ±11.4% viability). The percentage viability of MCF-7 cells cultivated in the presence of mats containing 5A8Q approximated that of free 5A8Q (45.0 ± 5.0%). The aqueous solutions of the complexes of 5A8Q showed slightly higher cytotoxic activity compared with that of the complexes of cr(PVA/CMC)/5A8Q mats for 72 h (Figure 8d). Approx. 12.9% and approx. 28.6% of MCF -7 cells preserved their viability when exposed for 72 h to Cu^2+^ and Fe^3+^ complexes of 5A8Q, respectively.

In contrast to both types of cancer cells, normal human HaCaT keratinocytes showed a lower decrease in their viability after exposure to cr(PVA/CMC)/5A8Q mats and their complexes (Figure 8e,f). After 24h the percentage of viable HaCaT cells was 90.5 ± 2.0%, 78.3 ± 6.8% and 83.2 ± 3.9% after treatment with cr(PVA/CMC)/5A8Q, cr(PVA/CMC)/5A8Q,Cu^2+^ and cr(PVA/CMC)/5A8Q,Fe^3+^ fibrous materials, respectively (Figure 8e). These percentages decreased with increasing exposure time and, at the 72nd hour, reached values of 82.2 ± 1.2% for cr(PVA/CMC)/5A8Q mats, 63.6 ± 4.3% for Cu^2+^ complexes and 65.1 ± 2.8% for Fe^3+^ complexes of cr(PVA/CMC)/5A8Q mats, respectively (Figure 8f). Thus, complexes had higher cytotoxicity towards normal keratinocytes than cr(PVA/CMC)/5A8Q mats. At the 72nd hour, free 5A8Q and its complexes reduced more strongly the viability of normal HaCaT cells than that of fibrous materials containing 5A8Q and their complexes (Figure 8f). The results obtained from the cell viability assay indicated that fibrous mats containing 5A8Q and their Cu^2+^ and Fe^3+^ complexes caused a significant decrease in cancer cell viability, while exhibiting lower cytotoxicity towards normal cells.

### 3.9. Fluorescence AO and EtBr Double Staining and DAPI Staining for Detection of Apoptosis

**Figure 9:**
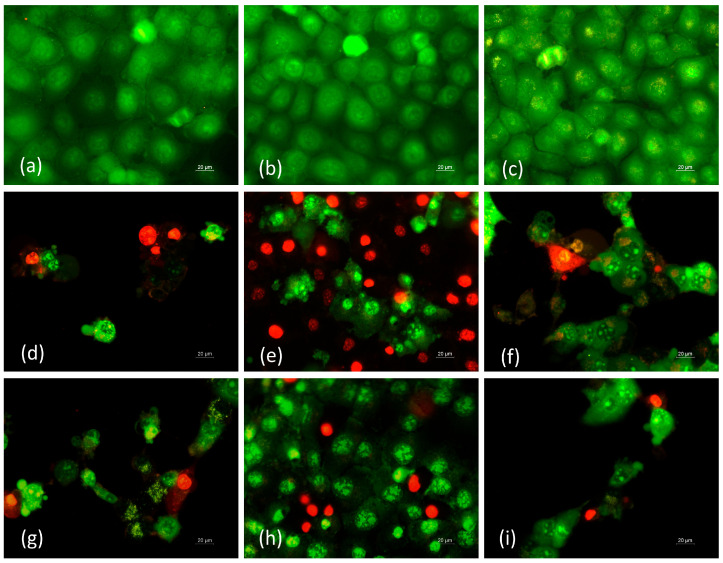
Fluorescence microscopy analysis of HeLa cancer cells incubated in the presence of different mats or solutions for 24 h and double-stained with AO/EtBr. (**a**) Untreated HeLa cells, (**b**) crPVA mat, (**c**) cr(PVA/CMC) mat, (**d**) cr(PVA/CMC)/5A8Q mat, (**e**) cr(PVA/CMC)/5A8Q,Cu^2+^ mat, (**f**) cr(PVA/CMC)/5A8Q,Fe^3+^ mat, (**g**) aqueous solution of 5A8Q, (**h**) solution of 5A8Q,Cu^2+^, (**i**) solution of 5A8Q,Fe^3+^, scale bar = 20 µm. All formulations containing 5A8Q and complexes were studied at a concentration of 5A8Q 20 µM.

AO/EtBr staining was performed to detect the morphological changes in cells exposed to cr(PVA/CMC)/5A8Q mats and their complexes with Cu^2+^ or Fe^3+^ ions. This fluorescence method allows easy discrimination between dead and viable cells. Viable cells are stained uniformly in green, early apoptotic cells are bright yellow–green stained with green–yellow fragments in the cytoplasm, while late apoptotic cells are orange–red stained, with condensation and aggregation of the chromatin, blabbing of the plasmatic membrane, nuclear fragmentation and formation of apoptotic bodies. Necrotic cells are red-stained, with no preserved membrane. Cell cultures of the tested tumor and non-tumor cell lines, treated with crPVA and cr(PVA/CMC) mats did not show any obvious morphological changes. In these cases, similar to the control cells, a dense monolayer of uniformly green stained cells was detected (Figure 9a–c; Supplementary Material, Figure S9a–c and Figure S11a–c. As a result of the treatment with cr(PVA/CMC)/5A8Q mats and their complexes and with solutions of 5A8Q and its complexes, HeLa cancer cells displayed the most distinct alterations, while non-cancer HaCaT cells appeared to be less affected (Figure 9d–i and Supplementary Material, Figure S9d–i and Figure S11d–i. The most significant changes in Hela cells were found after the exposure to cr(PVA/CMC)/5A8Q mats and their Fe^3+^ complexes and solutions of 5A8Q and its Fe^3+^ complex (Figure 9d,f,g,i). Cells with morphological changes typical of early apoptosis (green-stained cells with bright orange regions of condensed or fragmented chromatin in the nucleus) and late apoptosis (cells with a uniformly bright orange–red stained nucleus, blabbing of cytoplasmic membrane and cell fragmentation into membrane-bound apoptotic bodies) were observed. Impaired monolayer growth and reduction in cell number was also detected. Similar morphological alterations were found in MCF-7 cells exposed to cr(PVA/CMC)/5A8Q mats and their complexes and to solutions of 5A8Q and its complexes, but cells with signs of early apoptosis predominated (Supplementary Material, Figure S9d–i). The most considerable alterations in MCF-7 cells were observed after their incubation with the cr(PVA/CMC)/5A8Q,Cu^2+^ mat and with 5A8Q,Cu^2+^ (Supplementary Material, Figure S9e,h). As shown in the Supplementary Material (Figure S11d–f), normal HaCaT keratinocytes showed fewer morphological changes, mainly of the early-apoptotic type, after incubation with the studied mats containing 5A8Q and their complexes. No alterations were observed in normal keratinocytes after incubation with cr(PVA/CMC)/5A8Q mats and these keratinocytes were similar in morphology to the control cells (Supplementary Material, Figure S11a,d).

The apoptosis-inducing ability of the tested materials was also investigated by microscopic analysis of DAPI-stained cells (Supplementary Material, Figures S8 and S10). The activation of programmed cell death is associated with the appearance of specific morphological alterations in cells. Typically, cap-shaped chromatin margination is one of the early signs of apoptosis. During apoptosis, DNA condenses, a process that does not occur during necrosis, and this condensation can be used to distinguish apoptotic cells from live cells or necrotic cells. Figures S8 and S10 (Supplementary Material) present nuclei of untreated control cells and of cells exposed to formulations containing 5A8Q and their complexes. The nuclei of the respective control cells were generally spherical and had evenly distributed chromatin (Supplementary Material, Figures S8a and S10a). Cells treated with crPVA or cr(PVA/CMC) mats also showed unchanged nuclei without signs of apoptosis, as well as nuclei in a division phase similar to the control untreated cells (Supplementary Material, Figures S8b,c and S10b,c). HeLa cells treated with cr(PVA/CMC)/5A8Q mats and their complexes and with solutions of 5A8Q and its complexes demonstrated typical signs of apoptosis, i.e., significant nuclear shrinkage, condensation and fragmentation of chromatin (Supplementary Material, Figure S8d–i). The most pronounced changes in HeLa cell nuclei were seen after culturing in the presence of cr(PVA/CMC)/5A8Q mats and their Fe^3+^ complexes and of 5A8Q and its Fe^3+^ complex (Supplementary Material, Figure S8d,f,g,i). All tested formulations containing 5A8Q and their complexes also induced apoptotic alterations in the nuclear morphology of the MCF-7 cells, with the strongest effect observed in the cr(PVA/CMC)/5A8Q,Cu^2+^ mat and 5A8Q,Cu^2+^ (Supplementary Material, Figure S10e,h).

Figure S12 (Supplementary Material) demonstrates DAPI-stained untreated and treated HaCaT cells. The nuclei of HaCaT cells treated with crPVA and cr(PVA/CMC) mats were not changed and were morphologically similar to that of control cells (Supplementary Material, Figure S12b,c). The nuclei of normal keratinocytes incubated with cr(PVA/CMC)/5A8Q mats and their complexes and with tested solutions were altered to a lesser extent than those of cancer cells (Supplementary Material, Figure S12d–i). A slight condensation of chromatin, as well as fragmentation of the nucleus, was observed in treated cells; these changes were more pronounced after incubation with the solutions of 5A8Q and its complexes (Supplementary Material, Figure S12d–i). It should be noted that the nuclei of HaCaT cells exposed to a cr(PVA/CMC)/5A8Q mat demonstrated undamaged morphology as well as the presence of mitotic nuclei similar to the control cells (Supplementary Material, Figure S12d).

The results obtained from the fluorescence microscopy study demonstrated that mats containing 5A8Q and their complexes possessed higher cytotoxicity towards cancer HeLa and MCF-7 cells compared with non-cancer HaCaT cells; these results were in agreement with data from the cell viability test. The detected significant morphological alterations of the carcinoma cells exposed to the fabricated fibrous materials revealed that these materials caused cancer cell death via apoptosis.

## 4. Conclusions

Novel PVA/CMC fibrous mats loaded with 5A8Q were developed by one-pot electrospinning. Their complexes with Cu^2+^ and Fe^3+^ were also prepared. The morphology and structural and thermal properties of the obtained materials were analyzed. The obtained materials showed good stability in PBS (pH 7.4) for 24 h and 72 h. It was shown that the release of 5A8Q and its complexes from the mats containing 5A8Q and 5A8Q,Cu^2+^ (Fe^3+^) was mainly dependent on the hydrophilic/hydrophobic characteristics of the materials. The obtained 5A8Q-loaded cr(PVA/CMC) fibrous materials and their complexes had good efficacy in inhibiting the growth of bacteria *S. aureus* and *E. coli* and fungi *C. albicans*. Well-defined sterile zones were detected around the 5A8Q-loaded mats and their complexes. Fibrous materials with incorporated 5A8Q and their Cu^2+^ and Fe^3+^ complexes manifested high cytotoxicity towards human HeLa and MCF-7 cancer cells. The observed anticancer activity was due to the induction of apoptosis. The inhibitory effect of these materials was greater against cancer cells than against non-cancer HaCaT keratinocytes. These results indicate that the obtained novel fibrous materials have potential as dressing materials in wound treatment, as well as for application in the local therapy of cervical and breast cancer.

## Data Availability

The data presented in this study are available on request from the corresponding author.

## Supplementary Materials

The following supporting information can be downloaded at: https://www.mdpi.com/article/10.3390/polym15143140/s1, Figure S1. SEM micrographs and fiber diameter distribution of electrospun fibrous materials of: (a) PVA, (b) PVA/CMC (9:1 *w*/*w*), (c) PVA/CMC (8:2 *w*/*w*) and (d) PVA/CMC (7:3 *w*/*w*). Magnification ×5000; Figure S2. Mapping of elements C, O, N, Cl and Na, SEM micrograph (a) and EDX spectrum (b) of cr(PVA/CMC)/5A8Q fibrous materials; Figure S3. Mapping of elements C, O, N, Cl, Cu and Na, SEM micrograph (a) and EDX spectrum (b) of cr(PVA/CMC)/5A8Q,Cu^2+^ fibrous materials; Figure S4. Mapping of elements C, O, N, Cl, Na and Fe, SEM micrograph (a) and EDX spectrum (b) of cr(PVA/CMC)/5A8Q,Fe^3+^ fibrous materials; Figure S5. ATR-FTIR spectra of: (a) 5A8Q, (b) PVA/CMC mat and (c) PVA/CMC/5A8Q mat; Figure S6. XPS peak fittings for cr(PVA/CMC)/5A8Q (5wt% 5A8Q) mat ((a) C_1s_, (b) O_1s_, (c) N_1s_, (d) Na_1s_, (e) Cl_2p_); Figure S7. XPS peak fittings for cr(PVA/CMC)/5A8Q,Cu^2+^ (5wt% 5A8Q) mat ((a) C_1s_, (b) O_1s_, (c) N_1s_, (d) Cu_2p3/2_, (e) Cl_2p_, (f) Na_1s_) and cr(PVA/CMC)/5A8Q,Fe^3+^ (5wt% 5A8Q) mat ((g) C_1s_, (h) O_1s_, (i) N_1s_, (j) Fe_2p_, (k) Cl_2p_, (l) Na_1s_); Figure S8. Fluorescence microscopic images of HeLa cancer cells stained with DAPI: (a) untreated HeLa cells (control), (b) crPVA mat, (c) cr(PVA/CMC) mat, (d) cr(PVA/CMC)/5A8Q mat, (e) cr(PVA/CMC)/5A8Q,Cu^2+^ mat, (f) cr(PVA/CMC)/5A8Q,Fe^3+^ mat, (g) aqueous solution of 5A8Q, (h) solution of 5A8Q,Cu^2+^, (i) solution of 5A8Q,Fe^3+^, scale bar = 20 µm. All 5A8Q-containing formulations and their complexes were studied at a concentration of 5A8Q 20 µM; Figure S9. Fluorescence micrographs of AO/EtBr double-stained MCF-7 cancer cells incubated in the presence of different mats or solutions for 24 h. Cells after incubation with: (a) untreated MCF-7 cells, (b) crPVA mat, (c) cr(PVA/CMC) mat, (d) cr(PVA/CMC)/5A8Q mat, (e) cr(PVA/CMC)/5A8Q,Cu^2+^ mat, (f) cr(PVA/CMC)/5A8Q,Fe^3+^ mat, (g) aqueous solution of 5A8Q, (h) solution of 5A8Q,Cu^2+^, (i) solution of 5A8Q,Fe^3+^, scale bar = 20 µm. All 5A8Q-containing formulations and their complexes were studied at a concentration of 5A8Q 20 µM; Figure S10. Fluorescence microscopic images of MCF-7 cancer cells stained with DAPI: (a) untreated MCF-7 cells (control), (b) crPVA mat, (c) cr(PVA/CMC) mat, (d) cr(PVA/CMC)/5A8Q mat, (e) cr(PVA/CMC)/5A8Q,Cu^2+^ mat, (f) cr(PVA/CMC)/5A8Q,Fe^3+^ mat, (g) aqueous solution of 5A8Q, (h) solution of 5A8Q,Cu^2+^, (i) solution of 5A8Q,Fe^3+^, scale bar = 20 µm. All 5A8Q-containing formulations and their complexes were studied at a concentration of 5A8Q 20 µM; Figure S11. Fluorescence micrographs of AO/EtBr double-stained HaCaT cells incubated in the presence of different mats or solutions for 24 h. Cells after incubation with: (a) untreated HaCaT cells, (b) crPVA mat, (c) cr(PVA/CMC) mat, (d) cr(PVA/CMC)/5A8Q mat, (e) cr(PVA/CMC)/5A8Q,Cu^2+^ mat, (f) cr(PVA/CMC)/5A8Q,Fe^3+^ mat, (g) aqueous solution of 5A8Q, (h) solution of 5A8Q,Cu^2+^, (i) solution of 5A8Q,Fe^3+^, scale bar = 20 µm. All 5A8Q-containing formulations and their complexes were studied at a concentration of 5A8Q 20 µM; Figure S12. Fluorescence microscopic images of HaCaT cells stained with DAPI: (a) untreated HaCaT cells (control), (b) crPVA mat, (c) cr(PVA/CMC) mat, (d) cr(PVA/CMC)/5A8Q mat, (e) cr(PVA/CMC)/5A8Q,Cu^2+^ mat, (f) cr(PVA/CMC)/5A8Q,Fe^3+^ mat, (g) aqueous solution of 5A8Q, (h) solution of 5A8Q,Cu^2+^, (i) solution of 5A8Q,Fe^3+^, scale bar = 20 µm. All 5A8Q-containing formulations and their complexes were studied at a concentration of 5A8Q 20 µM; Table S1. Dynamic viscosity (η) and conductivity (σ) of the spinning solutions, mean fiber diameter (d) and porosity of the electrospun mats (P_mat_).
