# Supplementary material for: 5-Amino-8-hydroxyquinoline-containing Electrospun Materials Based on Poly(vinyl alcohol) and Carboxymethyl Cellulose and Their Cu2+ and Fe3+ Complexes with Diverse Biological Properties: Antibacterial, Antifungal and Anticancer
Source: Polymers (Basel). 2023 Jul 24;15(14):3140. doi: 10.3390/polym15143140 (PMC10383330; doi:10.3390/polym15143140)
Supplement: Supplementary file 1 [file polymers-15-03140-s001.zip › polymers-2500343-supplementary.pdf]

5-Amino-8-Hydroxyquinoline-Containing Electrospun Materials Based on Poly(Vinyl Alcohol) and Carboxymethyl Cellulose and Their Cu^{2+} and Fe^{3+} Complexes with Diverse Biological Properties: Antibacterial, Antifungal and Anticancer

Milena Ignatova [1,*], Nevena Manolova [1], Iliya Rashkov [1,*], Ani Georgieva [2], Reneta Toshkova [2] and Nadya Markova [3]

[1] Laboratory of Bioactive Polymers, Institute of Polymers, Bulgarian Academy of Sciences, Acad. G. Bonchev St., Bl. 103A, BG-1113 Sofia, Bulgaria; manolova@polymer.bas.bg
[2] Institute of Experimental Morphology, Pathology and Anthropology with Museum, Bulgarian Academy of Sciences, Acad. G. Bonchev St., Bl. 25, BG-1113 Sofia, Bulgaria; georgieva_any@abv.bg (A.G.); reneta.toshkova@gmail.com (R.T.)
[3] Institute of Microbiology, Bulgarian Academy of Sciences, Acad. G. Bonchev St., Bl. 26, BG-1113 Sofia, Bulgaria; markn@bas.bg
* Correspondence: ignatova@polymer.bas.bg (M.I.); rashkov@polymer.bas.bg (I.R.); Tel.: +359-(0)2-9792239 (M.I.); Fax: +359-(0)2-8700309 (M.I. & I.R.)

Synthesis of the 5A8Q,Cu^{2+} (Fe^{3+})

To synthesize the 5A8Q,Cu^{2+}, an ethanol solution of $CuCl_2$ (0.058 g, 0.43 mmol; 10 mL) was added to an ethanol solution of 5A8Q (0.1 g, 0.43 mmol; 20 mL). The reaction mixture was stirred for 12h at 25 °C. The 5A8Q,Fe^{3+} was synthesized using a procedure similar to that utilized for the preparation of the Cu^{2+} complex. The prepared ethanol solutions were cast on Petri dishes and dried under vacuum.

Assessment of the MIC of 5A8Q and 5A8Q,Cu^{2+}(Fe^{3+})

The minimum inhibitory concentration (MIC) of 5A8Q and 5A8Q,Cu^{2+}(Fe^{3+}) was estimated for *S. aureus* 749, *E. coli* 3588 and *C. albicans* 74, respectively. Both *S. aureus* and *E. coli* strains were cultivated overnight in Tryptic Soy Agar (TSA, Becton Dickinson, Heidelberg, Germany) at 37◦C. *C. albicans* was grown in Sabouraud Dextrose Agar (SDA, Becton Dickinson, Sparks, MD, USA) for 48 h at room temperature. A suspension of the bacteria *S. aureus* and *E. coli* was obtained in Tryptic Soy broth, and in Sabouraud Dextrose Broth for fungi *C. albicans*. After optical standardization the suspensions were diluted with fresh media to 1.2×10^6 cells/ml. The 5A8Q was dissolved in distilled water and 5A8Q,Cu^{2+}(Fe^{3+})) was dissolved in DMSO/distilled water at initial concentration of 5A8Q of 0.002 g/ml. Serial two fold dilutions of the tested compounds were prepared in the respective broths and transferred in tubes. Each tube contained 1.0 ml of compound dilution and 1.0 ml of bacterial or fungal suspension. Tubes with *S. aureus* and *E. coli* were incubated at 37◦C for 24 h, and those with *C. albicans* at room temperature for 48 h. Readings were taken by visually comparing the turbidity of each tube with that of the control tube (which was devoid of 5A8Q and its complexes).

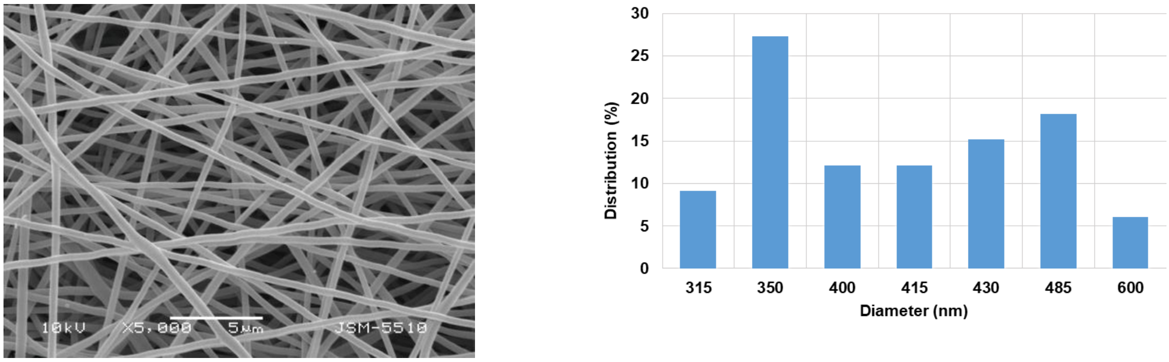

(**a**)

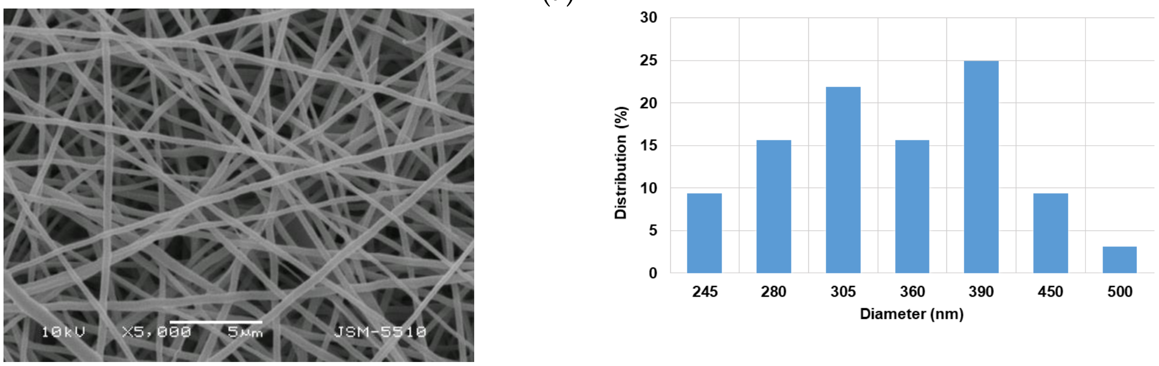

(**b**)

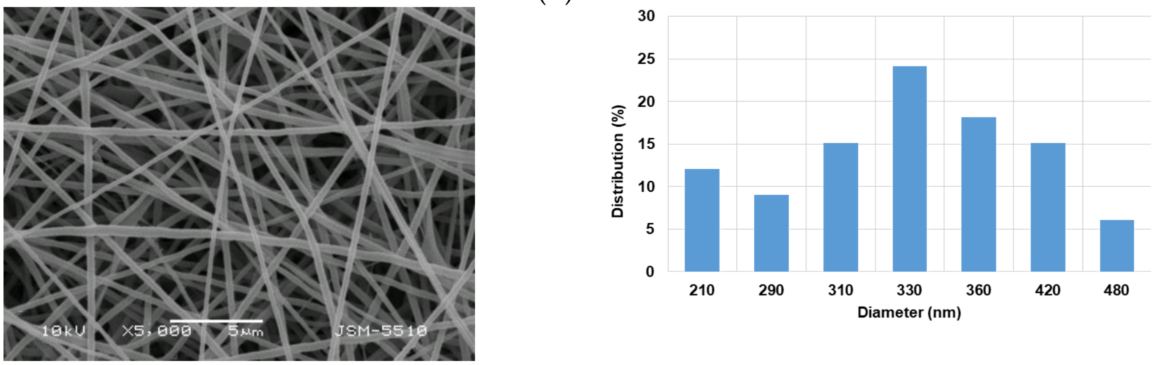

(**c**)

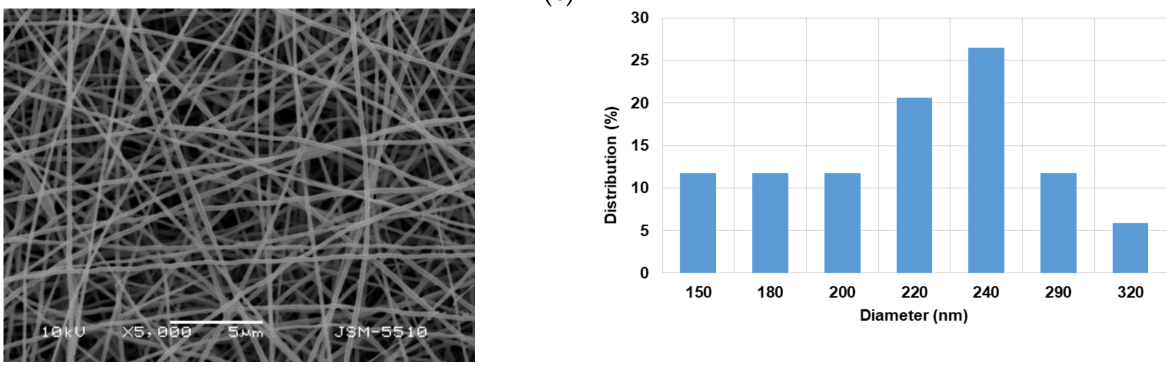

(**d**)

Figure S1. SEM micrographs and fiber diameter distribution of electrospun fibrous materials of: (**a**) PVA, (**b**) PVA/CMC (9:1 w/w), (**c**) PVA/CMC (8:2 w/w) and (**d**) PVA/CMC (7:3 w/w). Magnification ×5000.

Table S1. Dynamic viscosity (η) and conductivity (σ) of the spinning solutions, mean fiber diameter (d), and porosity of the electrospun mats (P_{mat}).

Electrospun mats	(cP)	(μS/cm)	d (nm)	P_{mat} (%)
PVA	1390	1620	412 ± 80	55.0 ± 0.8
PVA/CMC (9/1 w/w)	940	1970	345 ± 80	57.0 ± 1.0
PVA/CMC (8/2 w/w)	890	2130	340 ± 80	64.0 ± 1.2
PVA/CMC (7/3 w/w)	610	3254	225 ± 47	67.0 ± 1.8
PVA/CMC/5A8Q (3 wt% 5A8Q)	855	2150	330 ± 80	59.0 ± 2.0
PVA/CMC/5A8Q (5 wt% 5A8Q)	790	2200	324 ± 80	56.0 ± 1.5

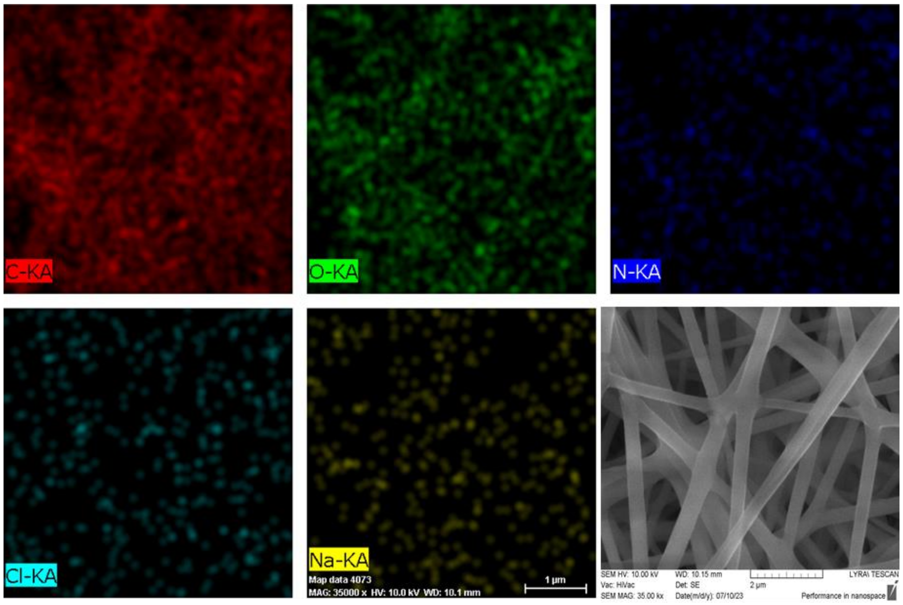

(**a**)

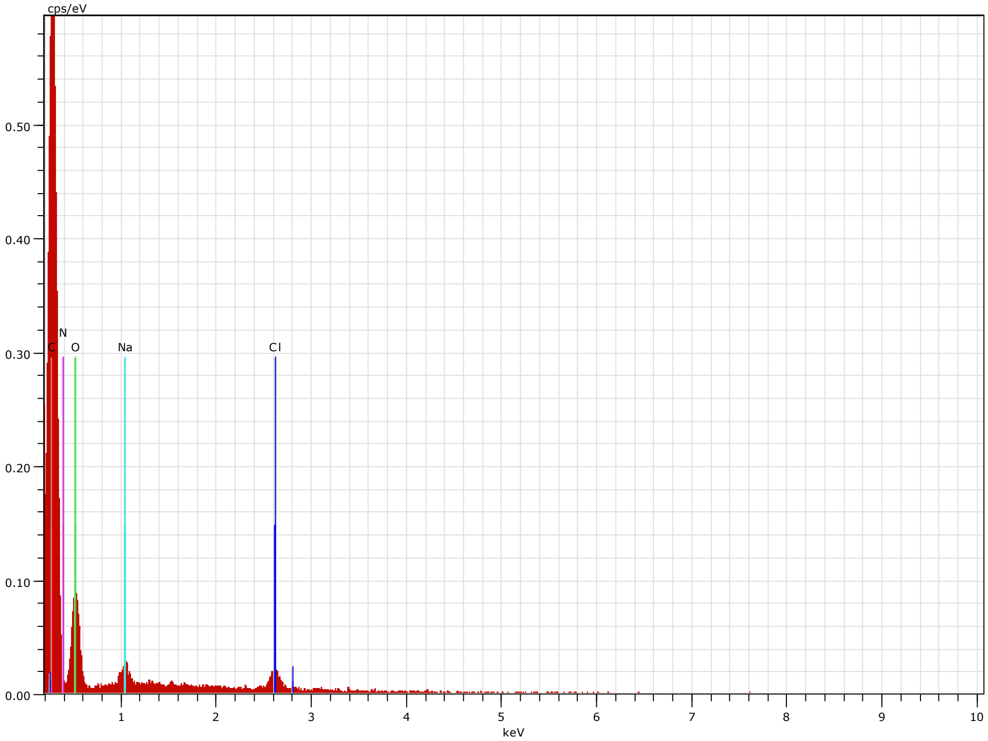

(**b**)

Figure S2. Mapping of elements C, O, N, Cl and Na, SEM micrograph (**a**) and EDX spectrum (**b**) of cr(PVA/CMC)/5A8Q fibrous materials.

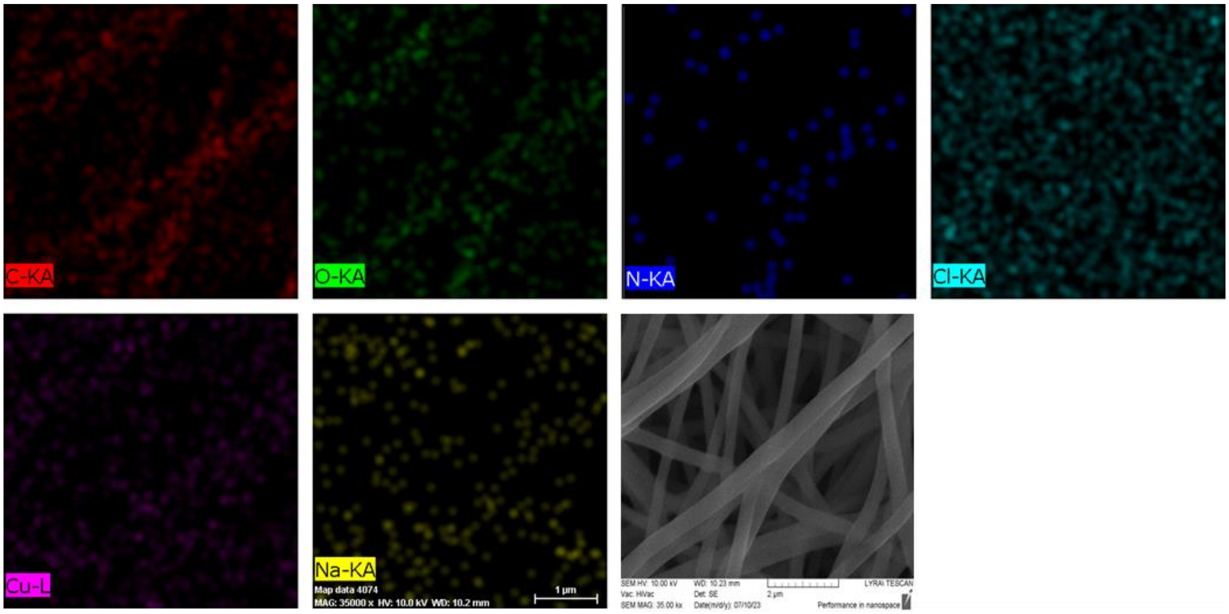

(**a**)

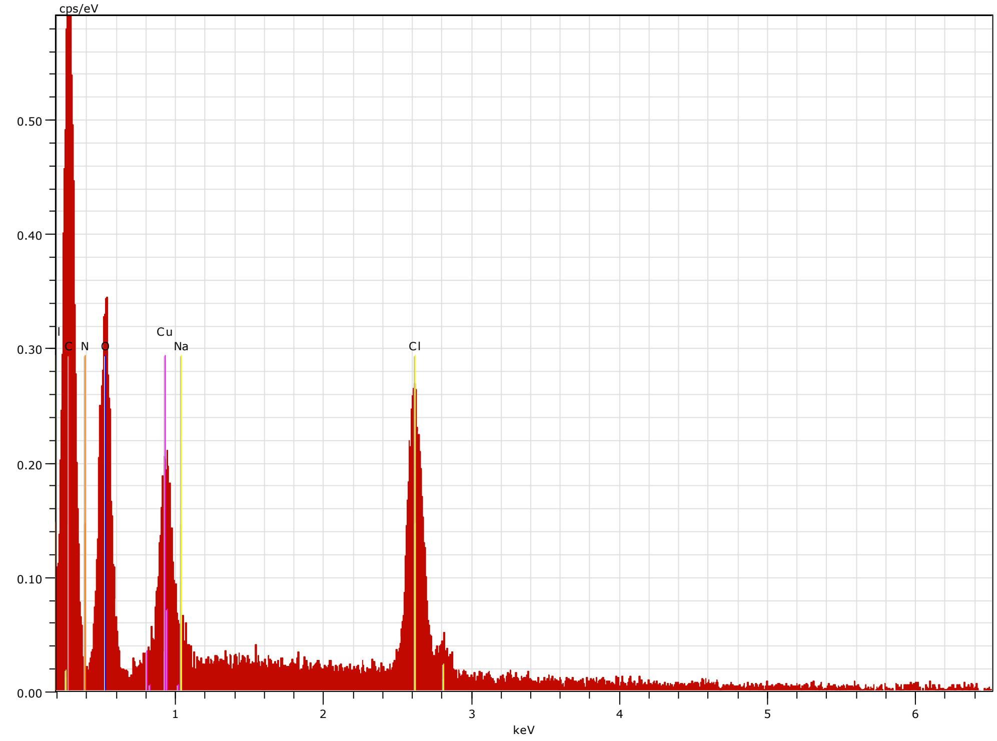

(**b**)

Figure S3. Mapping of elements C, O, N, Cl, Cu and Na, SEM micrograph (**a**) and EDX spectrum (**b**) of cr(PVA/CMC)/5A8Q,Cu^{2+} fibrous materials.

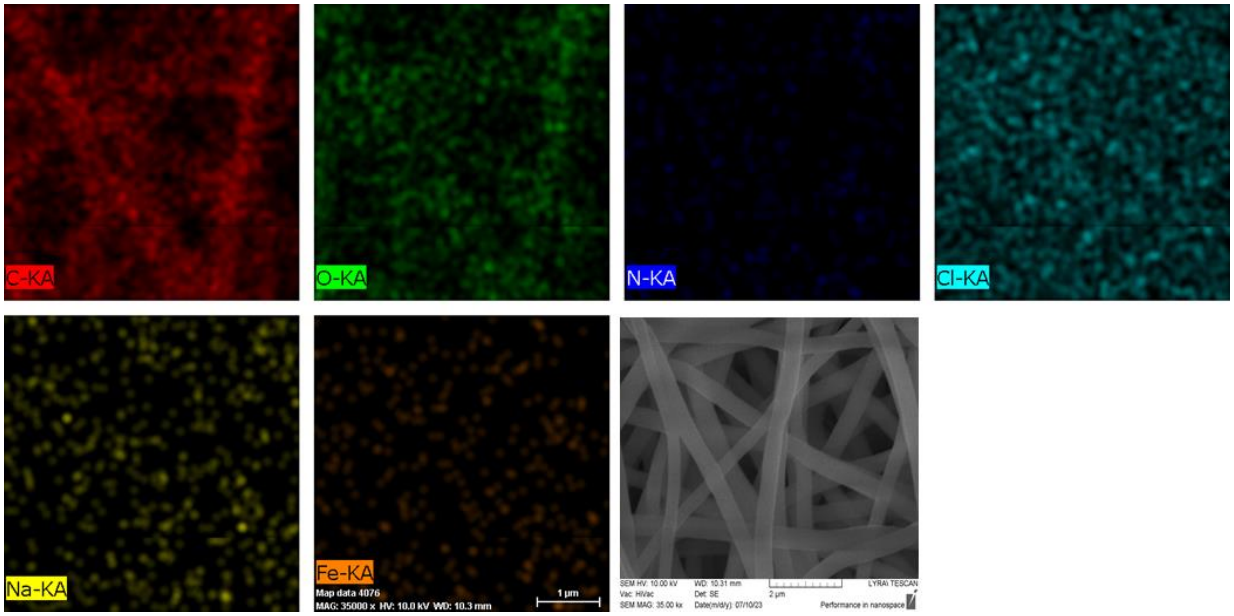

(**a**)

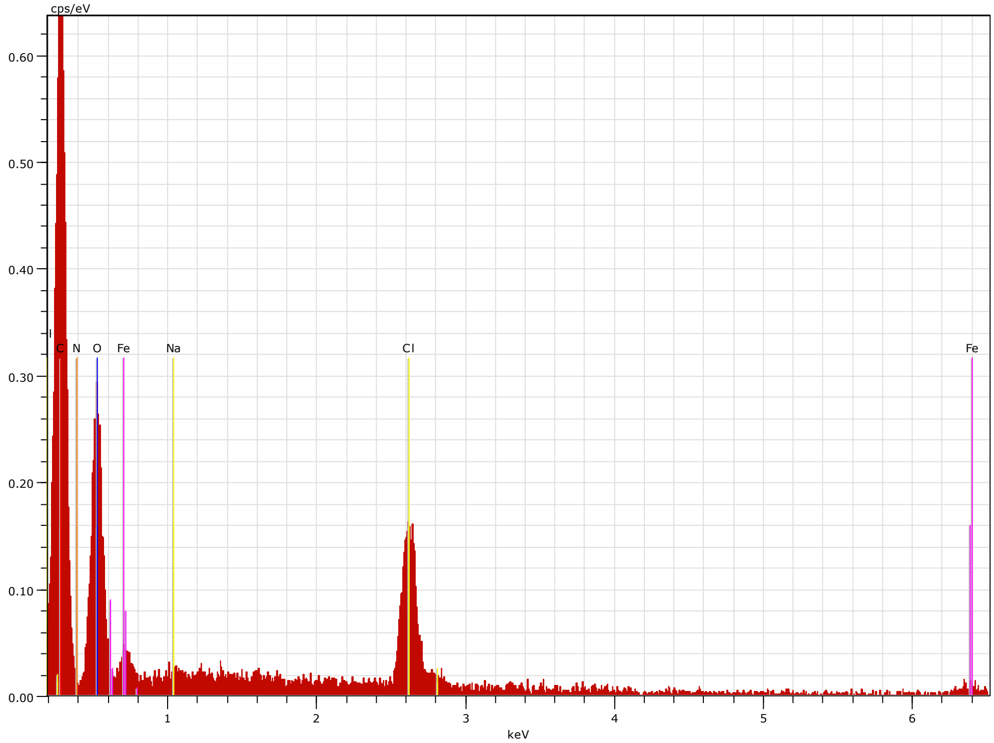

(**b**)

Figure S4. Mapping of elements C, O, N, Cl, Na and Fe, SEM micrograph (**a**) and EDX spectrum (**b**) of cr(PVA/CMC)/5A8Q,Fe^{3+} fibrous materials.

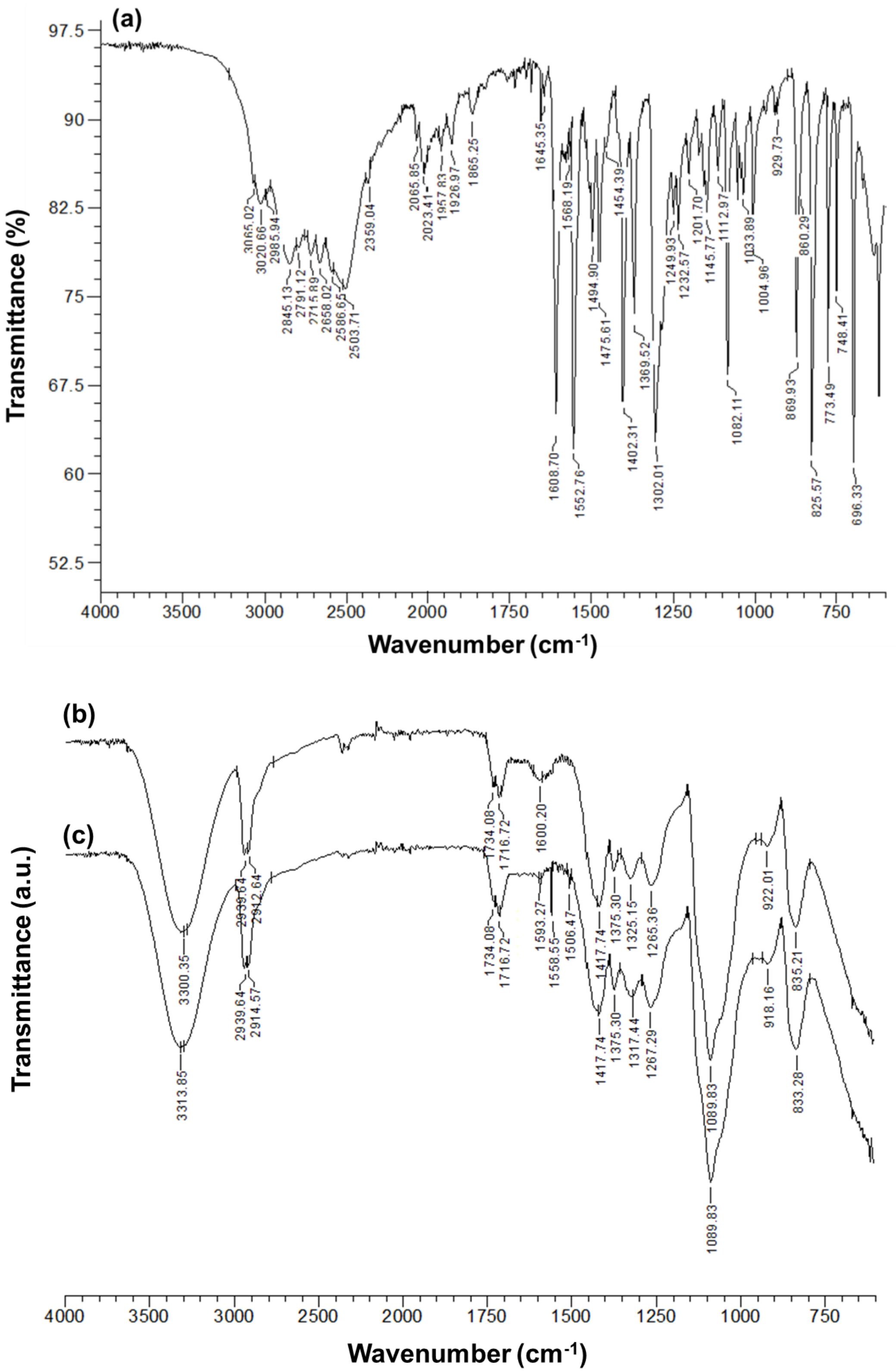

Figure S5. ATR-FTIR spectra of: (**a**) 5A8Q, (**b**) PVA/CMC mat and (**c**) PVA/CMC/5A8Q mat.

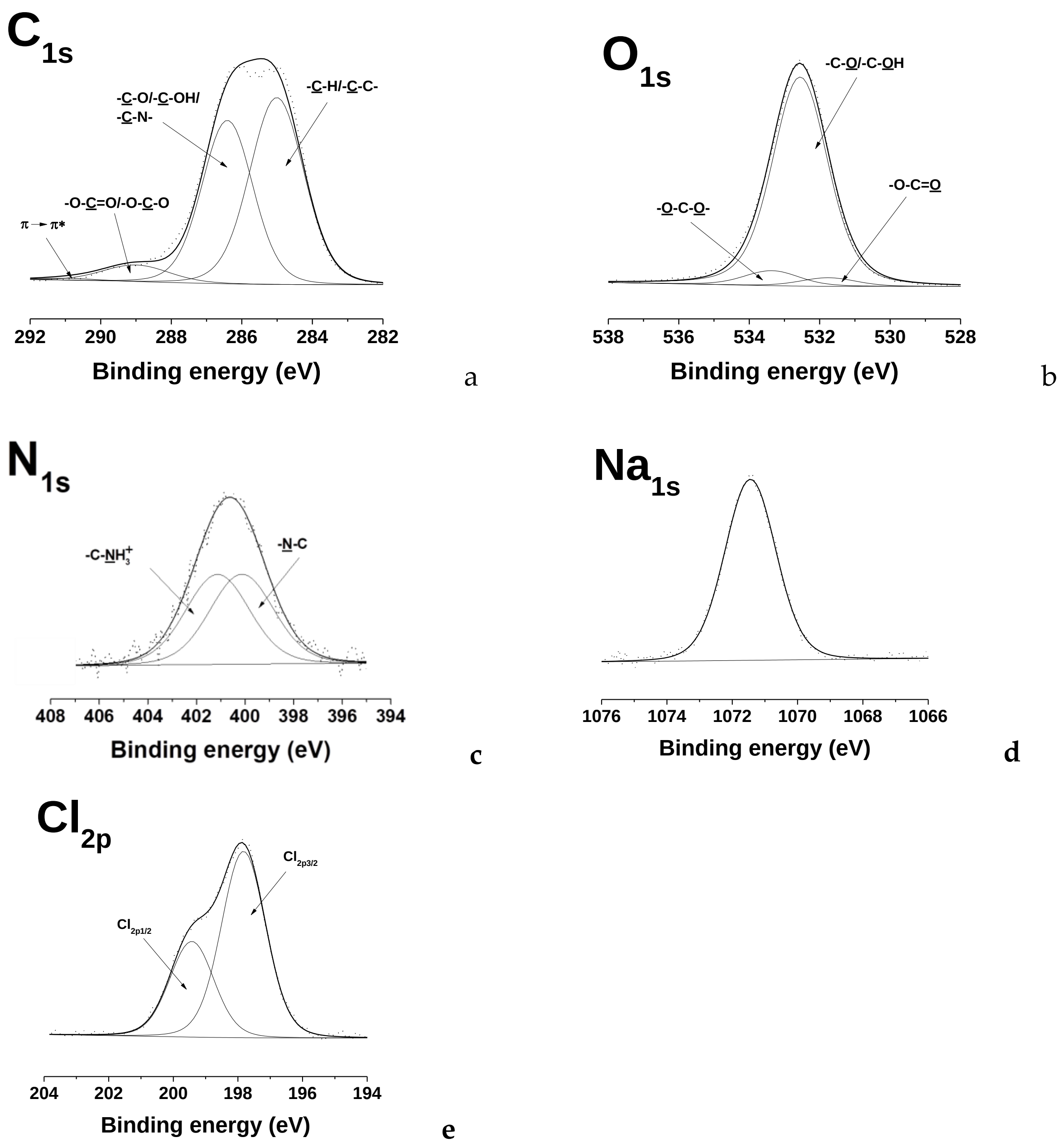

Figure S6. XPS peak fittings for cr(PVA/CMC)/5A8Q (5wt% 5A8Q) mat [(**a**) C_{1s}, (**b**) O_{1s}, (**c**) N_{1s}, (**d**) Na_{1s}, (**e**) Cl_{2p}].

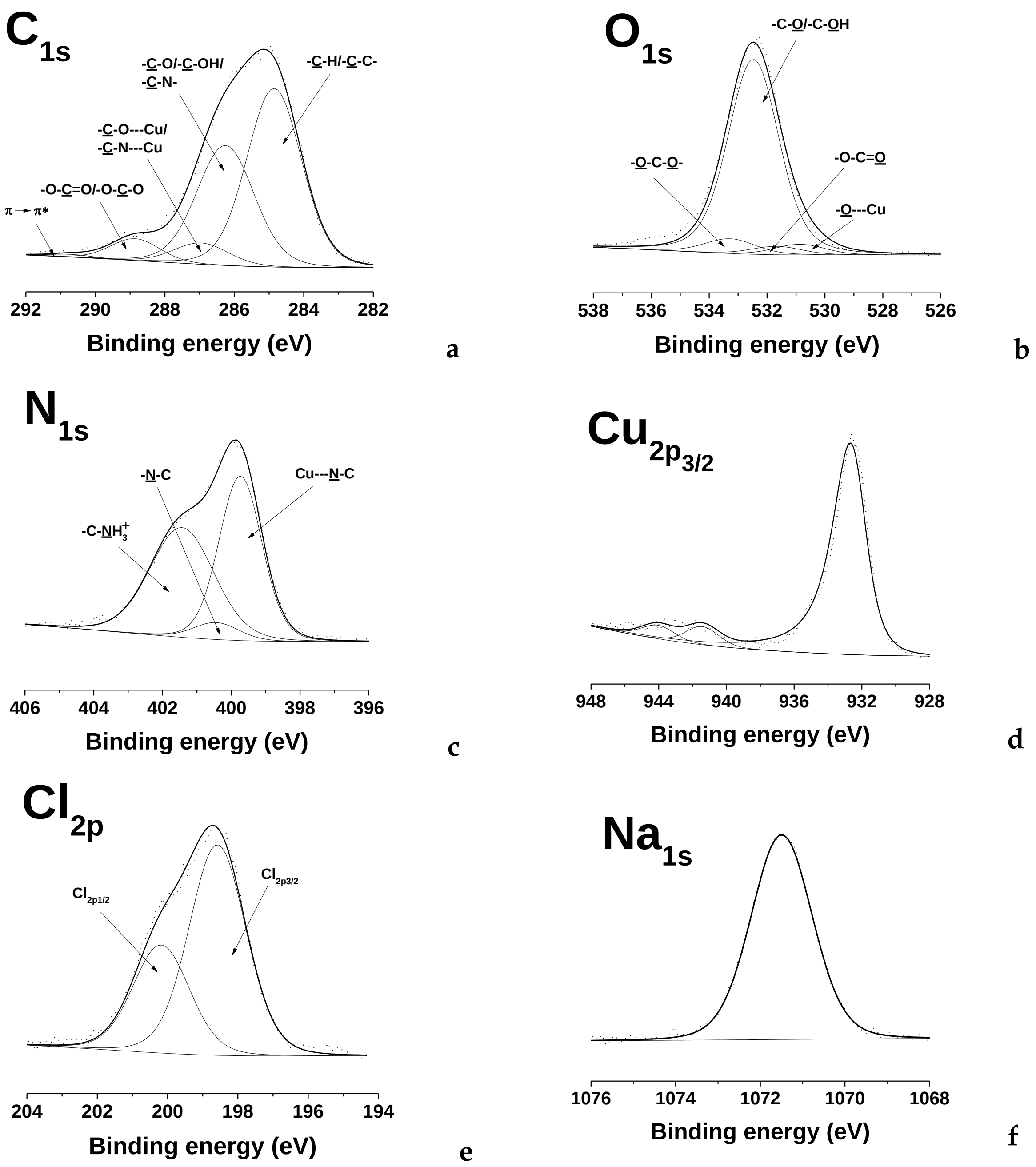
C1s
-C-O/-C-OH/
-C-N-
-C-H/-C-C-
-C-O---Cu/
-C-N---Cu
-O-C=O/-O-C-O
π → π*
292 290 288 286 284 282
Binding energy (eV)
a
O1s
-C-O/-C-OH
-O-C-O-
-O-C=O
-O---Cu
538 536 534 532 530 528 526
Binding energy (eV)
b
N1s
-N-C
Cu---N-C
-C-NH3+
406 404 402 400 398 396
Binding energy (eV)
c
Cu2p3/2
948 944 940 936 932 928
Binding energy (eV)
d
Cl2p
Cl2p1/2
Cl2p3/2
204 202 200 198 196 194
Binding energy (eV)
e
Na1s
1076 1074 1072 1070 1068
Binding energy (eV)
f

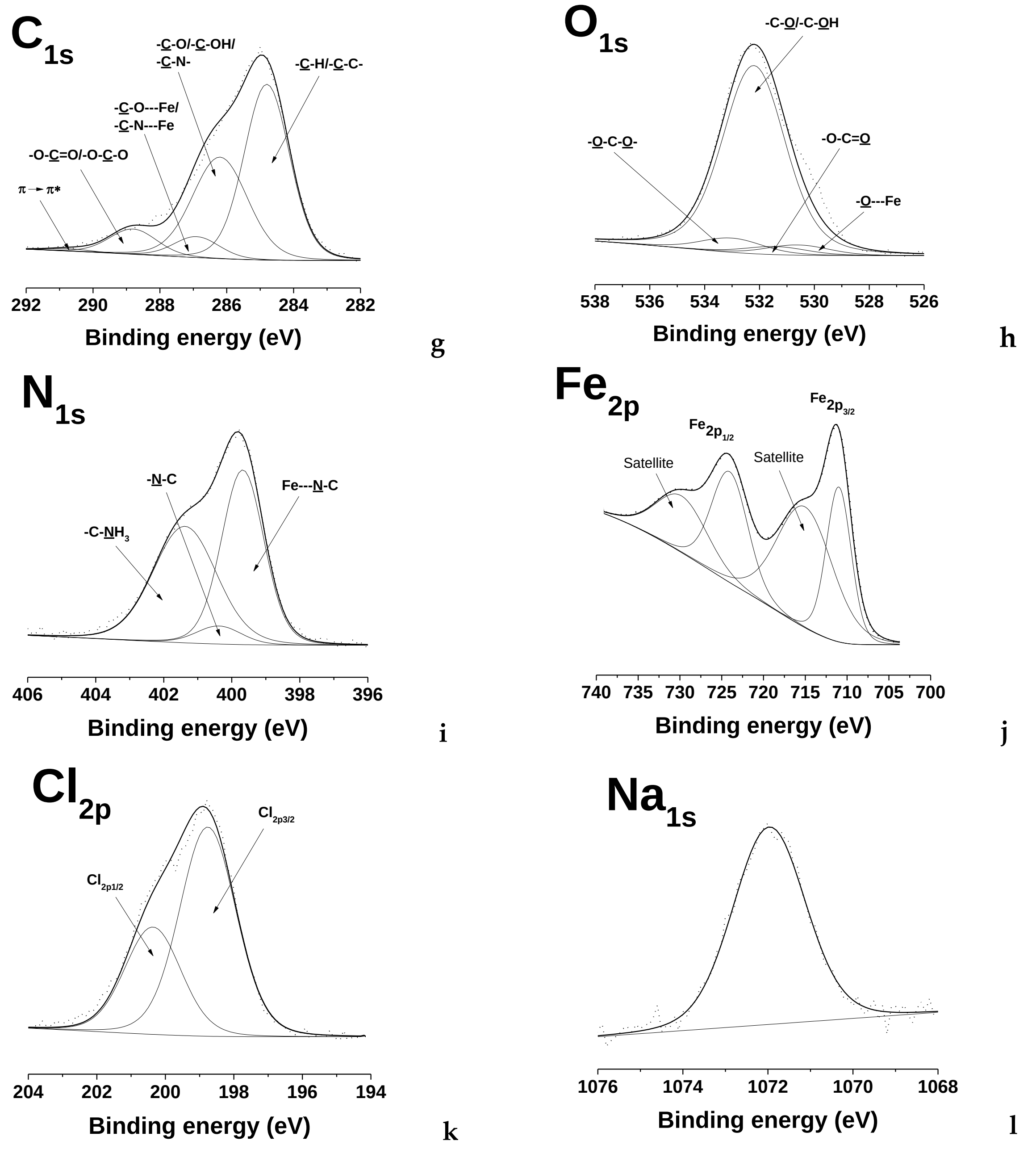

Figure S7. XPS peak fittings for cr(PVA/CMC)/5A8Q,Cu^{2+} (5wt% 5A8Q) mat [(**a**) C_{1s}, (**b**) O_{1s}, (**c**) N_{1s}, (**d**) $Cu_{2p3/2}$, (**e**) Cl_{2p}, (**f**) Na_{1s}] and cr(PVA/CMC)/5A8Q,Fe^{3+} (5wt% 5A8Q) mat [(**g**) C_{1s}, (**h**) O_{1s}, (**i**) N_{1s}, (**j**) Fe_{2p}, (**k**) Cl_{2p}, (**l**) Na_{1s}].

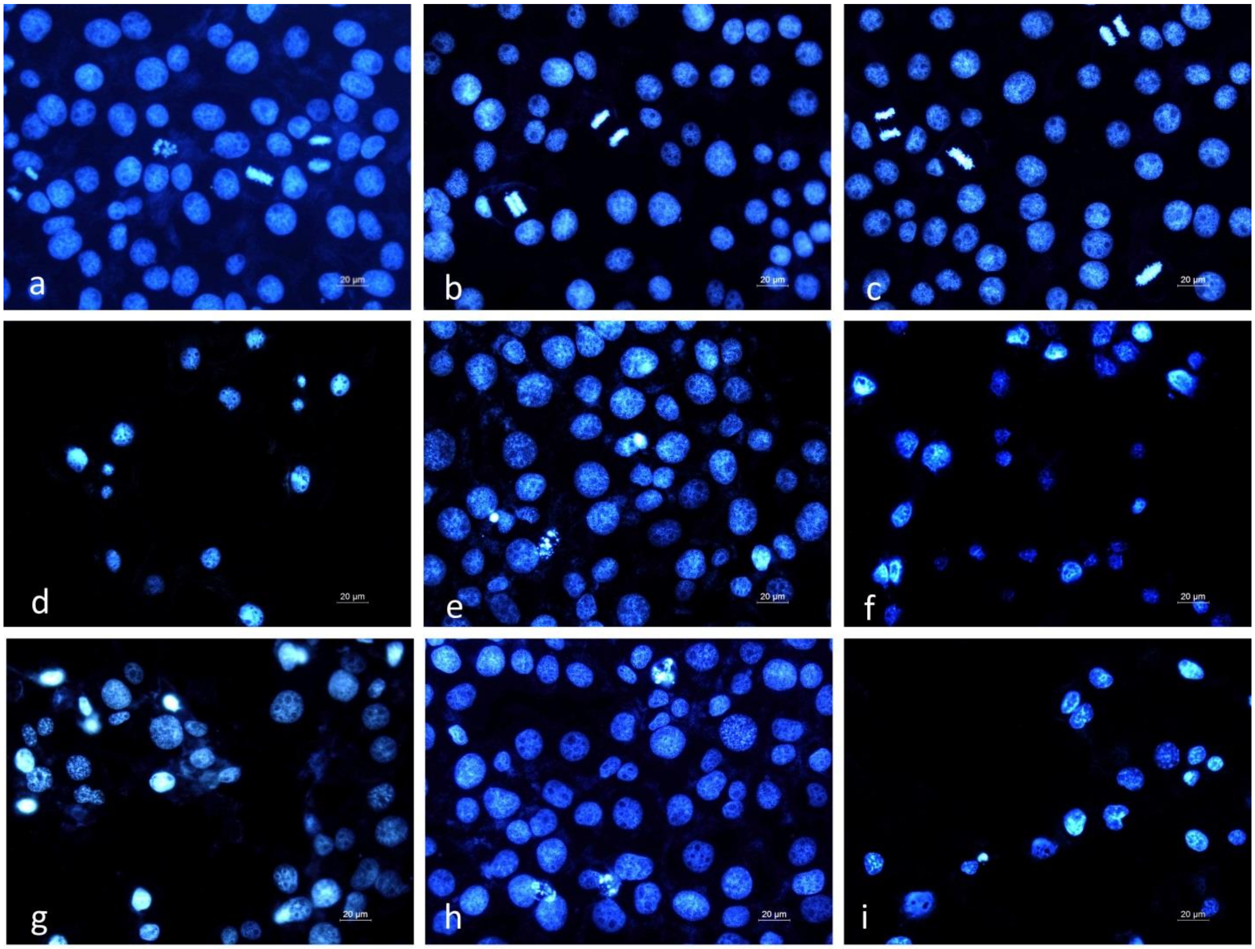

Figure S8. Fluorescence microscopic images of HeLa cancer cells stained with DAPI: (**a**) untreated HeLa cells (control), (**b**) crPVA mat, (**c**) cr(PVA/CMC) mat, (**d**) cr(PVA/CMC)/5A8Q mat, (**e**) cr(PVA/CMC)/5A8Q,Cu^{2+} mat, (**f**) cr(PVA/CMC)/5A8Q,Fe^{3+} mat, (**g**) aqueous solution of 5A8Q, (**h**) solution of 5A8Q,Cu^{2+}, (**i**) solution of 5A8Q,Fe^{3+}, scale bar = 20 μm. All 5A8Q-containing formulations and their complexes were studied at a concentration of 5A8Q 20 μM.

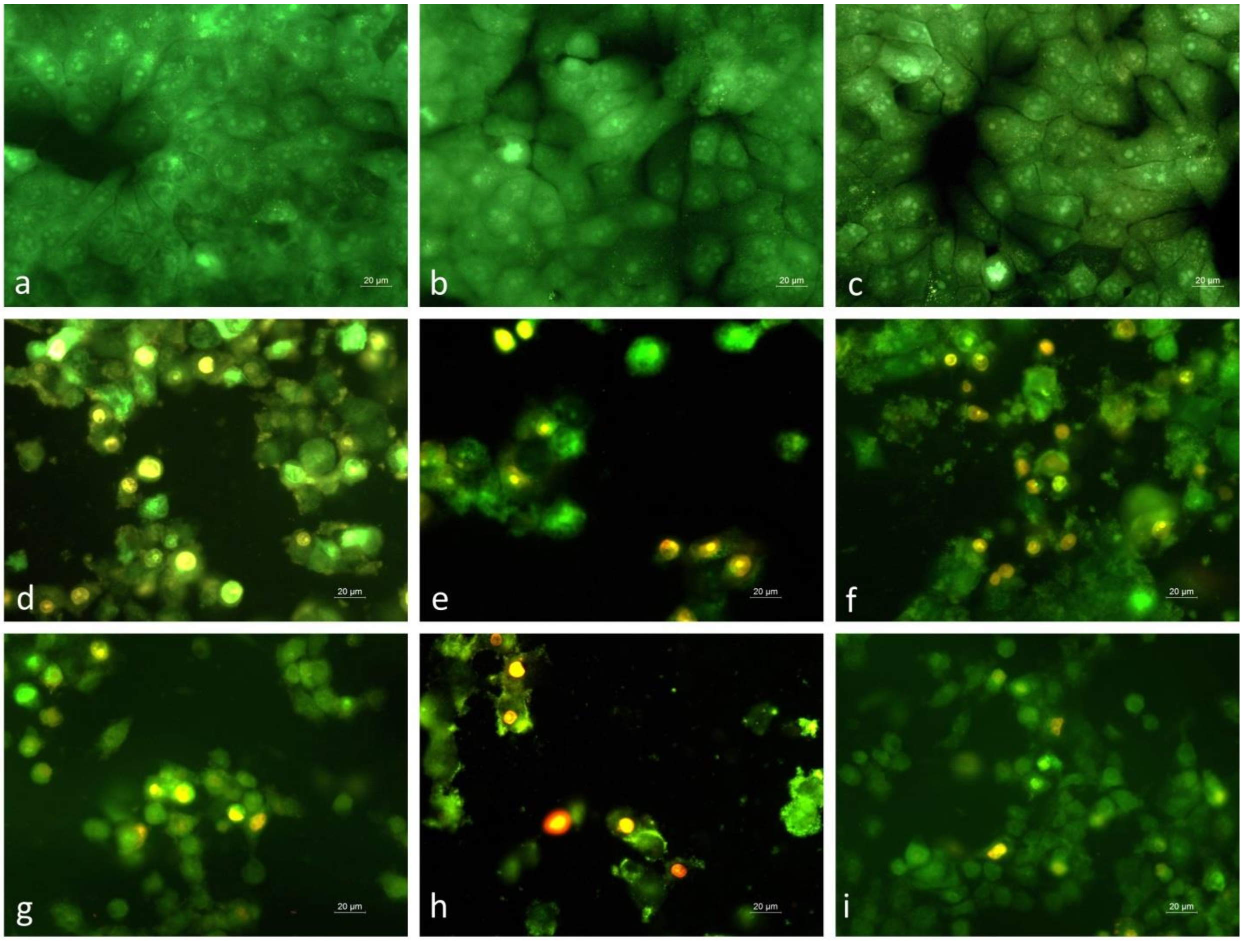

Figure S9. Fluorescence micrographs of AO/EtBr double-stained MCF-7 cancer cells incubated in the presence of different mats or solutions for 24 h. Cells after incubation with: (**a**) untreated MCF-7 cells, (**b**) crPVA mat, (**c**) cr(PVA/CMC) mat, (**d**) cr(PVA/CMC)/5A8Q mat, (**e**) cr(PVA/CMC)/5A8Q,Cu^{2+} mat, (**f**) cr(PVA/CMC)/5A8Q,Fe^{3+}mat, (**g**) aqueous solution of 5A8Q, (**h**) solution of 5A8Q,Cu^{2+}, (**i**) solution of 5A8Q,Fe^{3+}, scale bar = 20 μm. All 5A8Q-containing formulations and their complexes were studied at a concentration of 5A8Q 20 μM.

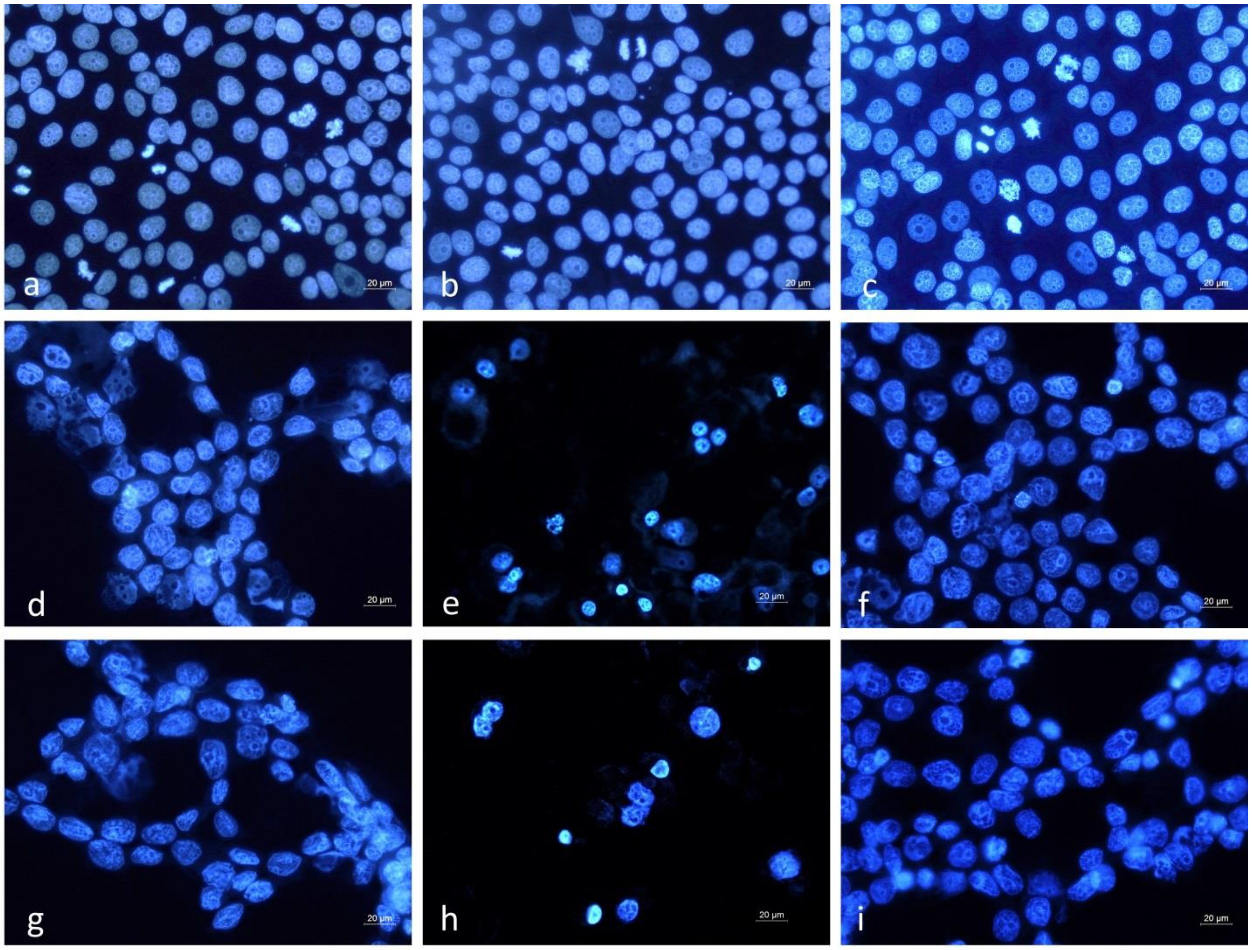

Figure S10. Fluorescence microscopic images of MCF-7 cancer cells stained with DAPI: (**a**) untreated MCF-7 cells (control), (**b**) crPVA mat, (**c**) cr(PVA/CMC) mat, (**d**) cr(PVA/CMC)/5A8Q mat, (**e**) cr(PVA/CMC)/5A8Q,Cu^{2+} mat, (**f**) cr(PVA/CMC)/5A8Q,Fe^{3+} mat, (**g**) aqueous solution of 5A8Q, (**h**) solution of 5A8Q,Cu^{2+}, (**i**) solution of 5A8Q,Fe^{3+}, scale bar = 20 µm. All 5A8Q-containing formulations and their complexes were studied at a concentration of 5A8Q 20 µM.

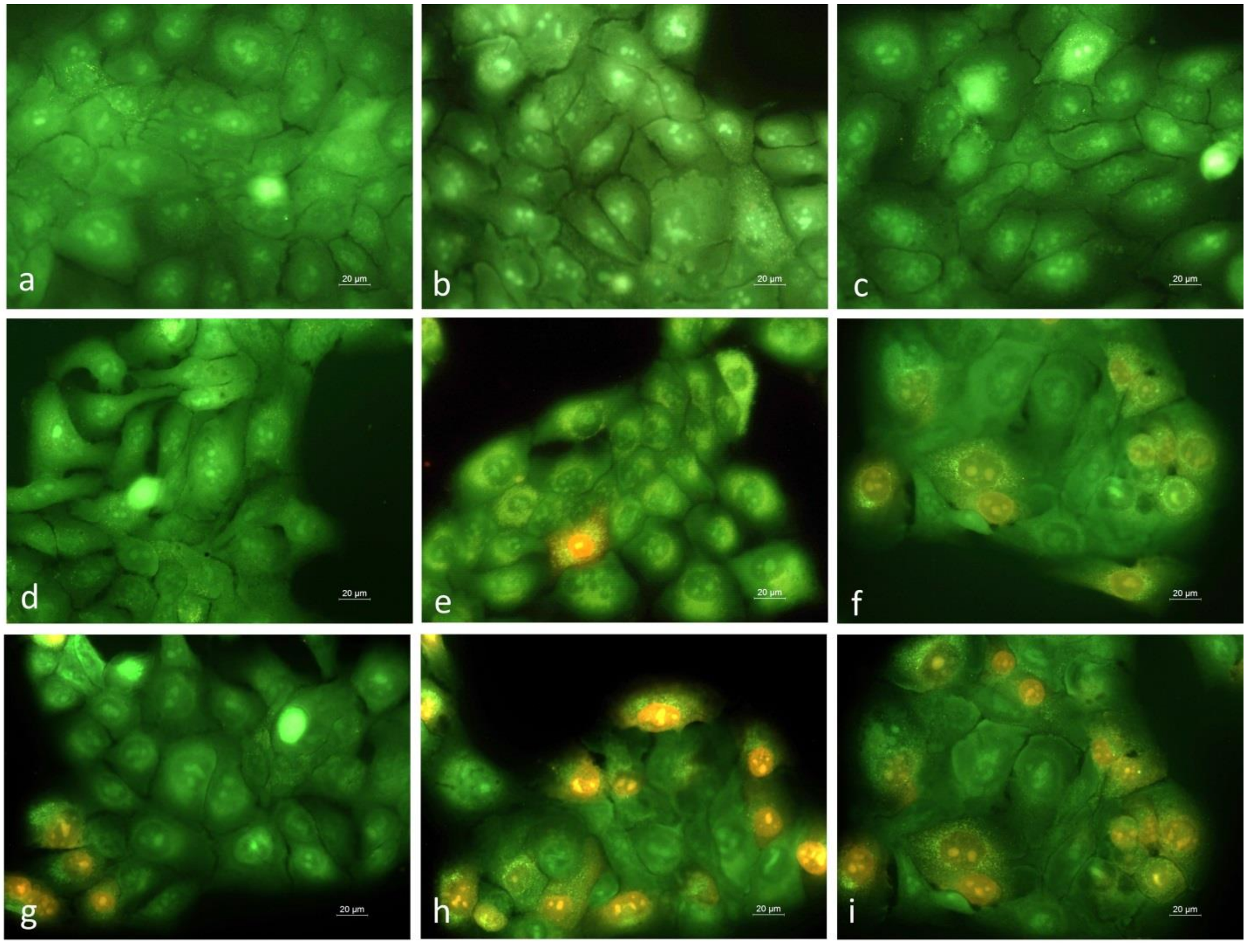

Figure S11. Fluorescence micrographs of AO/EtBr double-stained HaCaT cells incubated in the presence of different mats or solutions for 24 h. Cells after incubation with: (**a**) untreated HaCaT cells, (**b**) crPVA mat, (**c**) cr(PVA/CMC) mat, (**d**) cr(PVA/CMC)/5A8Q mat, (**e**) cr(PVA/CMC)/5A8Q,Cu^{2+} mat, (**f**) cr(PVA/CMC)/5A8Q,Fe^{3+}mat, (**g**) aqueous solution of 5A8Q, (**h**) solution of 5A8Q,Cu^{2+}, (**i**) solution of 5A8Q,Fe^{3+}, scale bar = 20 μm. All 5A8Q-containing formulations and their complexes were studied at a concentration of 5A8Q 20 μM.

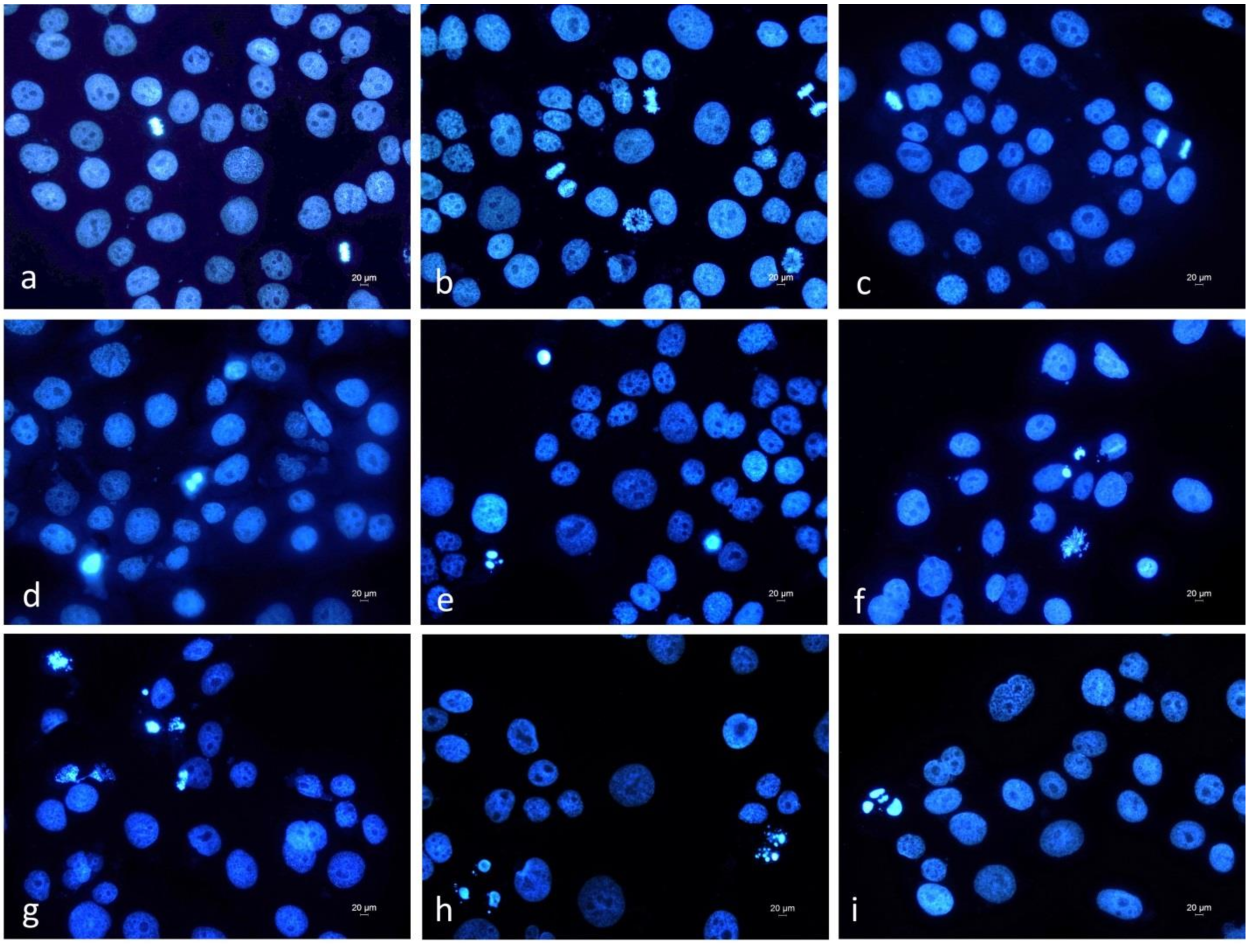

Figure S12. Fluorescence microscopic images of HaCaT cells stained with DAPI: (**a**) untreated HaCaT cells (control), (**b**) crPVA mat, (**c**) cr(PVA/CMC) mat, (**d**) cr(PVA/CMC)/5A8Q mat, (**e**) cr(PVA/CMC)/5A8Q,Cu^{2+} mat, (**f**) cr(PVA/CMC)/5A8Q,Fe^{3+} mat, (**g**) aqueous solution of 5A8Q, (**h**) solution of 5A8Q,Cu^{2+}, (**i**) solution of 5A8Q,Fe^{3+}, scale bar = 20 μm. All 5A8Q-containing formulations and their complexes were studied at a concentration of 5A8Q 20 μM.
